# PET/CT of breast cancer regional nodal recurrences: an evaluation of contouring atlases

**DOI:** 10.1186/s13014-020-01576-6

**Published:** 2020-06-01

**Authors:** Laura Beaton, Luminita Nica, Scott Tyldesley, Kenny Sek, Gareth Ayre, Maria Aparicio, Lovedeep Gondara, Caroline Speers, Alan Nichol

**Affiliations:** 1 Department of Radiation Oncology, BC Cancer, Vancouver Centre, Vancouver, British Columbia Canada; 2 Cancer Surveillance and Outcomes, BC Cancer, Vancouver Centre, Vancouver, British Columbia Canada; 3 Department of Nuclear Medicine, BC Cancer, Vancouver Centre, Vancouver, British Columbia Canada

## Abstract

**Background:**

To validate the Radiation Therapy Oncology Group (RTOG) and European Society for Radiotherapy and Oncology (ESTRO) breast cancer nodal clinical target volumes (CTVs) and to investigate the Radiotherapy Comparative Effectiveness Consortium (RADCOMP) Posterior Neck volume in relation to regional nodal recurrences (RNR).

**Methods:**

From a population-based database, 69 patients were identified who developed RNR after curative treatment for breast cancer. RNRs were detected with 18-fluorodeoxyglucose-positron emission tomography-computed tomography (PET/CT). All patients were treatment-naïve for RNR when imaged. The RTOG and ESTRO nodal CTVs and RADCOMP Posterior Neck volumes were contoured onto a template patient’s CT. RNRs were contoured on each PET/CT and deformed onto the template patient’s CT. Each RNR was represented by a 5 mm diameter epicentre, and categorized as ‘inside’, ‘marginal’ or ‘outside’ the CTV boundaries.

**Results:**

Sixty-nine patients with 226 nodes (median 2, range 1–11) were eligible for inclusion. Thirty patients had received adjuvant tangent and regional nodal radiotherapy, 16 tangent-only radiotherapy and 23 no adjuvant radiotherapy. For the RTOG CTVs, the RNR epicentres were 70% (158/226) inside, 4% (8/226) marginal and 27% (60/226) outside. They included the full extent of the RNR epicentres in 38% (26/69) of patients. Addition of the RADCOMP Posterior Neck volume increased complete RNR coverage to 48% (33/69) of patients. For the ESTRO CTVs, the RNR epicentres were 73% (165/226) inside, 2% (4/226) marginal and 25% (57/226) outside. They included the full extent of the RNR epicentres in 57% (39/69) of patients. Addition of the RADCOMP Posterior Neck volume increased complete RNR coverage to 70% (48/69) of patients.

**Conclusions:**

The RTOG and ESTRO breast cancer nodal CTVs do not fully cover all potential areas of RNR, but the ESTRO nodal CTVs provided full coverage of all RNR epicentres in 19% more patients than the RTOG nodal CTVs. With addition of the RADCOMP Posterior Neck volume to the ESTRO CTVs, 70% of patients had full coverage of all RNR epicentres.

## Introduction

Regional nodal relapse (RNR) is an important predictor of breast cancer-specific (BCSS) and overall survival [1]. It is well established that adjuvant breast/chest wall radiotherapy (RT) reduces the risk of relapse and improves overall survival [1, 2]. Furthermore, for high-risk patients, RT to the regional lymph nodes improves local control, reduces distant metastases, and improves overall survival [1, 3–5]. More recently, data from the EORTC 22922-10925, MA.20, and DBCG-IMN trials showed a benefit of regional nodal irradiation (RNI) even in early-stage breast cancer [6–8]. As a result, an increasing number of patients are receiving adjuvant RNI. In this context, it is imperative to cover the volume containing microscopic disease without irradiating unnecessarily large nodal clinical target volumes (CTVs). Furthermore, the effort to better map RNRs is particularly important as the use of intensity-modulated RT (IMRT) and proton beam therapy for breast cancer becomes more common.

In 2009, the Radiation Therapy Oncology Group (RTOG) published a consensus guideline to improve the accuracy of nodal target delineation and reduce individual inconsistencies in contouring [9, 10]. In 2015, the European Society for Radiotherapy and Oncology (ESTRO) published a consensus guideline which differed from the RTOG atlas. Its purpose was to create a nodal CTV that matched the historical treatment volume of 3D conformal locoregional breast radiotherapy. Compared to the RTOG CTV, it added 0.5 cm lateral and medial margins on the IMC vessels, added an interpectoral nodal CTV between the pectoralis major and minor, and reduced the cranial border of the supraclavicular fossa (SCF) CTV from the cricoid cartilage to 0.5 cm cranial to the subclavian vein [11, 12]. Since then, a number of studies have evaluated the location of RNRs in relation to the RTOG Breast Cancer Atlas [13–19] and the ESTRO Breast Cancer Atlas [17–21], noting that their supraclavicular CTVs have poor coverage of diseased posterior neck lymph nodes (Table 1). Some studies have mapped areas of RNR onto a template patient with the atlases already contoured [13, 15–20], but few have used deformable registration [19]. Furthermore, most of these prior studies identified RNRs with computerized tomography (CT), which is inferior to 18-fluorodeoxyglucose positron emission tomography/computed tomography (PET/CT) [23, 24]. Many studies included patients with de novo nodal disease, on the assumption that areas harboring gross disease at presentation are those same regions most likely to harbor microscopic disease needing adjuvant radiotherapy [13–16, 19]. However, we felt that a study examining RNR alone would provide valuable information about the greatest possible extent of microscopic disease at presentation. Thus, we set out to validate the RTOG and ESTRO nodal CTVs. The RADCOMP atlas was developed for breast cancer patients requiring locoregional RT in the RTOG 3510 phase III clinical trial of conventional RT versus proton RT [25]. We also wanted to explore the benefit of using the RADCOMP Posterior Neck volume for covering RNR outside of the RTOG and ESTRO supraclavicular nodal CTVs [25].

**Table 1:** Studies of regional nodal relapses in relation to nodal contouring atlases

	Total no. patients (nodes)	Inclusion criteria	No. relapsed patients (nodes)	Nodal area assessed	No. patients received adjuvant RNI	Imaging technique for relapse	Nodal mapping technique	Nodal contours or epicentres	Nodal Atlases Studied
Brown et al. 2015 [13]	62 (161)	De novo and relapse	18 (44)	SCF	4	CT/PET or MRI	Manual mapping Template patient	Epicentre	RTOG
Gentile et al. 2015 [14]	30 (309)	De novo only	0 (0)	Axilla	NA	CT scan	Rigid fusion Individual	Contours	RTOG
Jing et al. 2015 [15]	55 (524)	De novo and relapse	38 (NA)	SCF	3	CT scan or FDG/PET	Manual mapping Template patient	Epicentres + contours	RTOG
Jethwa et al. 2016 [16]	130 (67)	De novo and relapse	7 (15)	IMN	NA	CT, PET/CT or MRI	Manual mapping Template patient	Epicentres	RTOG
Chang et al. 2017 [17]	129 (235)	Relapse only	129 (235)	All	49	CT scan	Manual mapping Template patient	Epicentres	RTOG ESTRO
Chang et al. 2018 [18]	234 (337)	Relapse only	234 (337)	All	130	CT scan or PET/CT	Manual mapping Template patient	Epicentres	RTOG ESTRO
Borm et al. 2018 [19]	235 (580)	De novo and relapse	197 (410)	All	NA	PET/CT	Deformable registration Template patient	Contours	RTOG ESTRO
DeSelm et al. 2018 [20]	153 (243)	Relapse only	153 (243)	All	NA	CT, PET/CT or MRI	Manual mapping Template patient	Epicentres	RTOG ESTRO
Kowlaski et al. 2019 [22]	102 (389)	Not stated	Not stated	All	NA	PET/CT	Manual mapping Template patient	Epicentres	RTOG ESTRO RADCOMP
Almahariq et al. 2020 [21]	106 (107)	De novo only	0 (0)	Axilla Level I	NA	CT or PET, Biopsy Clip	Manual mapping Template patient	Epicentres	RTOG ESTRO RADCOMP
Current study	69 (226)	Relapse only	69 (226)	All	30	PET/CT	Deformable registration Template patient	Epicenters	RTOG ESTRO RADCOMP

## Materials and methods

### Patient population

Between July 2005 and March 2013, all patients treated curatively with a diagnosis of invasive breast cancer that had undergone PET/CT were identified from a population-based database. During this time period, contouring regional nodes for radiotherapy planning was not routine at our institution. All PET/CTs were performed for restaging at the time of clinically detected RNR. Patients were included if they had a diagnosis of RNR (defined as [^18^F] fluorodeoxyglucose (FDG)-avid tissue in ipsilateral regional lymph nodes) with or without distant metastatic relapse, and if FDG PET/CT was performed > 6 months after initial curative surgery (to exclude those likely to have had macroscopic disease during their curative treatment). Patients were excluded if macroscopic disease was detected at time of RT planning, if a new primary breast cancer was diagnosed, or if they had undergone any treatment for RNR prior to PET/CT to ensure we had a cohort of patients with unperturbed RNR. Patient, tumor and treatment details, as well as clinical outcomes were obtained from the Breast Cancer Outcomes Unit which maintains a prospectively collected database.

### Regional nodal mapping

The RTOG and ESTRO breast cancer nodal CTVs and the RADCOMP Posterior Neck volume were contoured by radiation oncologists (LB and AN) onto a radiotherapy planning CT of a template patient who was scanned on a 12.5-degree breast board. At the time of CTV contouring, LB and AN were blinded to the FDG uptake on the PET/CTs. The PET/CT showing RNR for each patient was loaded onto the MIM planning system (version 6.4, MIM Software Inc., Cleveland, OH). FDG-avid nodes were included as RNRs if they had FDG uptake on PET/CT with a corresponding lymph node on CT/PET and were reported as abnormal by a nuclear medicine physician. FDG-avid RNRs were contoured by a nuclear medicine specialist (KS) and radiation oncologist (LB) using the MIM PET Edge™ contour tool, a gradient-based technique that draws a threshold surface defined by the steepest drop off in standardized uptake values. The Edge tool generates reproducible contours that correspond to the anatomic size and location of the corresponding masses on CT [26]. Using the MIM software, a deformable registration algorithm was used to deform each contoured FDG-avid RNR onto the template patient’s planning CT, with the RTOG-CTV, ESTRO-CTV and RADCOMP Posterior Neck volume already outlined. The position of each deformed RNR was visually reviewed, and edited, if necessary, to ensure accurate localization. The spherical growth pattern of breast cancer metastases in regional lymph nodes is an established imaging feature that is used to distinguish between normal and diseased lymph nodes [27, 28]. Hence, on the understanding that large RNRs started growing from a small nidus of disease, a 5 mm sphere was created at the mathematical centroid of the RNR to represent the location of microscopic disease at presentation. Relapses were mapped according to whether the patient had received RNI, tangent only RT or no RT.

### Analysis of atlas coverage

Each RNR epicentre was assessed in relation to the contoured RTOG, ESTRO and RADCOMP Posterior Neck CTVs. Coverage for each RNR was defined as: ‘inside’, ‘marginal’ or ‘outside’. In keeping with previous trials, locations of RNR were defined as ‘inside’: entirely or mostly within the CTVs, ‘marginal’: mostly outside the CTV edges, and ‘outside’: completely outside the CTV contours [13]. For ‘marginal’ or ‘outside’ epicentres, the location of RNR relative to the CTVs was recorded.

### Statistical analyses

Descriptive statistics and analyses were performed using Fisher’s exact tests for continuous variables and χ^2^ test for categorical variables. Univariate logistic regression was used to assess the association of these baseline patient/tumor factors: number of positive nodes, grade, stage, lymphovascular invasion and use of adjuvant RNI. Two-sided *p*-values < 0.05 were considered statistically significant. Statistical analysis was performed using SAS version 9.3 (SAS Institute, Cary NC) and R 3.2.3. This study was reviewed and approved by the BC Cancer Research Ethics Board.

## Results

### Patient characteristics

**Fig. 1:**
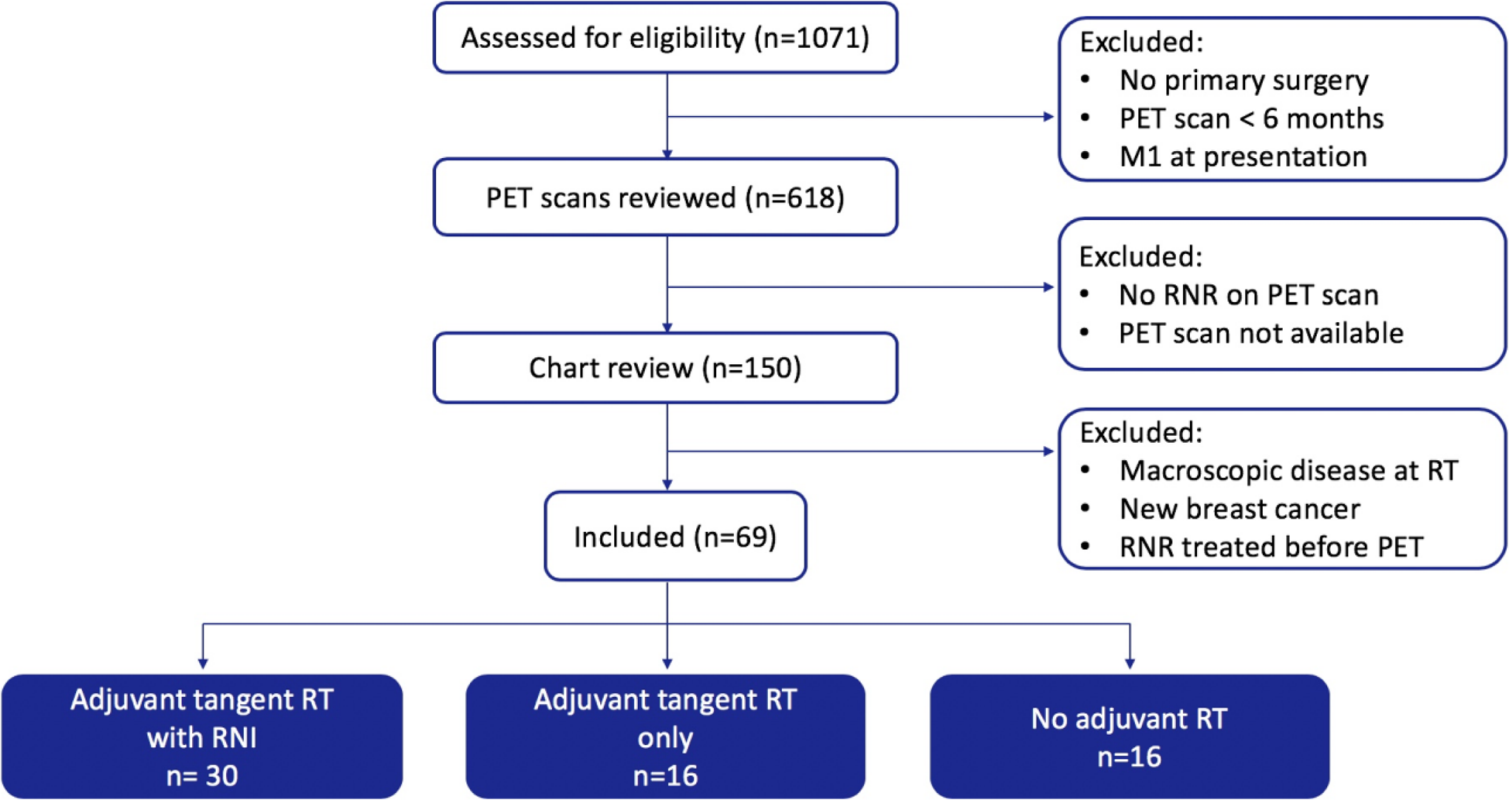
Study schema. *Abbreviations:* PET, 18-fluorodeoxyglucose-positron emission tomography-computed tomography; RNI, regional lymph node irraditaion; RT, radiotherapy; RNR, regional nodal relapse

Between 2005 and 2013, 1071 patients underwent PET/CT for breast cancer. Figure 1 details how our cohort of 69 eligible patients was selected. Baseline patient, tumor and treatment details, as well as relapse data are shown in Table 2. In total, 226 FDG-positive RNRs were identified in 69 patients (range 1–11, median 2 RNR/patient, mean 3.3 RNR/patient). Ninety-three percent (64/69) of patients had biopsies to confirm relapse: 28 of regional nodes, 22 of breast/chest wall and 14 of distant sites. Thirty-eight patients (55%) had distant metastatic disease at the time of RNR, whilst 31 patients (45%) had RNR without distant metastases. Regarding initial adjuvant treatment, 30 patients had 3/4-field RNI to the breast/chest wall region and the supraclavicular lymph nodes (including 28 patients with axillary RT and 12 patients with RT to the first three interspaces of the IMCs); 16 patients had tangent-only RT (including two patients who received wide-tangent RT covering the IMN chain without axillary/supraclavicular RT); and 23 patients had no RT.

**Table 2:** Patient and tumor baseline characteristics and locations of nodal relapses

		All patients ***N*** = 69	RNI ***N*** = 30	Tangent only ***N*** = 16	No RT ***N*** = 23	***P***-value
**Age** **Median (Range)**		49 (29–84)	48.5 (30–76)	52 (35–75)	48 (29–84)	0.45
**Laterality**	**Right**	34 (49%)	14 (47%)	8 (50%)	12 (52%)	0.92
	**Left**	35 (51%)	16 (53%)	8 (50%)	11 (48%)	
**Grade**	**1**	6 (9%)	1 (3%)	1 (6%)	4 (17%)	0.27
	**2**	27 (39%)	11 (37%)	8 (50%)	8 (35%)	
	**3**	32 (46%)	17 (57%)	6 (38%)	9 (40%)	
	**Unknown**	4 (4%)	1 (3%)	1 ((6%)	2 (9%)	
**Tumor size**	**0-2 cm**	32 (46%)	10 (33%)	11 (69%)	11 (48%)	0.03
	**> 2–5 cm**	26 (38%)	12 (40%)	5 (31%)	9 (40%)	
	**> 5 cm**	9 (14%)	8 (27%)	0 (0%)	1 (4%)	
	**Unknown**	2 (3%)	0 (0%)	0 (0%)	2 (9%)	
**Number of positive nodes**	**0**	32 (46%)	5 (17%)	14 (88%)	13 (57%)	< 0.01
	**1–3**	19 (28%)	13 (43%)	2 (12%)	4 (17%)	
	**≥ 4**	14 (20%)	11 (37%)	0 (0%)	3 (13%)	
	**Unknown**	4 (6%)	1 (3%)	0 (0%)	3 (13%)	
**Stage**	**I**	24 (35%)	6 (20%)	6 (38%)	12 (52%)	< 0.01
	**II**	28(40%)	16 (54%)	6 (38%)	6 (26%)	
	**III**	6(9%)	4 (13%)	0 (0%)	2 (9%)	
	**Unknown**	11(16%)	4 (13%)	4 (24%)	3 (13%)	
**Her2 status**	**Positive**	6 (9%)	4 (13%)	1 (6%)	1 (4%)	0.54
	**Negative**	40 (58%)	19(63%)	9 (56%)	12 (52%)	
	**Unknown**	23 (33%)	7 (23%)	6 (38%)	10 (44%)	
**Estrogen status**	**Positive**	47 (68%)	17 (57%)	12 (75%)	18 (78%)	0.11
	**Negative**	20 (29%)	13 (43%)	3 (19%)	4 (17%)	
	**Unknown**	2 (3%)	0 (0%)	1 (6%)	1 (4%)	
**Surgery**	**BCS**	31 (45%)	10 (33%)	13 (81%)	8 (35%)	< 0.01
	**Mastectomy**	38 (55%)	20 (66%)	3 (19%)	15 (65%)	
**Axillary lymph node dissection**	**Yes**	53 (77%)	27 (90%)	12 (75%)	14 (61%)	0.04
	**No**	16 (23%)	3 (10%)	4 (25%)	9 (31%)	
**Chemotherapy**	**Yes**	46 (67%)	23 (77%)	12 (75%)	11 (48%)	0.07
	**No**	23 (33%)	7 (23%)	4 (25%)	12 (52%)	
**Hormone therapy**	**Yes**	41 (59%)	16 (53%)	12 (75%)	13 (57%)	0.14
	**No**	28(41%)	14 (47%)	4 (25%)	10 (43%)	
**Isolated RNR** **RNR and distant metastases**		31 (45%)	9 (30%)	9 (56%)	13 (57%)	0.09
		38 (55%)	21 (70%)	7 (44%)	10 (43%)	
**Number of RNR**		226	92	48	86	
**RNR per Patient**		3.3	3.1	3.0	3.7	0.28
**RNR Locations**	**SCF** **(CTV4)**	35	20	7	8	
**(ESTRO CTV)**	**Axilla Level 1 (CTV1)**	64	22	15	27	
	**Axilla Level 2 (CTV2)**	27	7	8	12	
	**Interpectoral**	11	5	1	5	
	**Axilla Level 3 (CTV3)**	34	12	6	16	
	**IMN**	34	18	7	9	
	**RADCOMP** **Posterior Neck**	21	8	4	9	

*Abbreviation*: *RNR* Regional nodal recurrence

### Coverage of regional nodes at relapse

For the RTOG CTVs, the RNR epicentres were 70% (158/226) inside, 4% (8/226) marginal and 27% (60/226) outside. They included the full extent of the RNR epicentres in 38% (26/69) of patients. Addition of the RADCOMP Posterior Neck volume increased complete RNR coverage to 48% (33/69) of patients. For the ESTRO-CTVs, the RNR epicentres were 73% (165/226) inside, 2% (4/226) marginal and 25% (57/226) outside. They included the full extent of the RNR epicentres in 57% (39/69) of patients. Addition of the RADCOMP Posterior Neck volume increased complete RNR coverage to 70% (48/69) of patients.

### Location of regional nodes at relapse

**Fig. 2:**
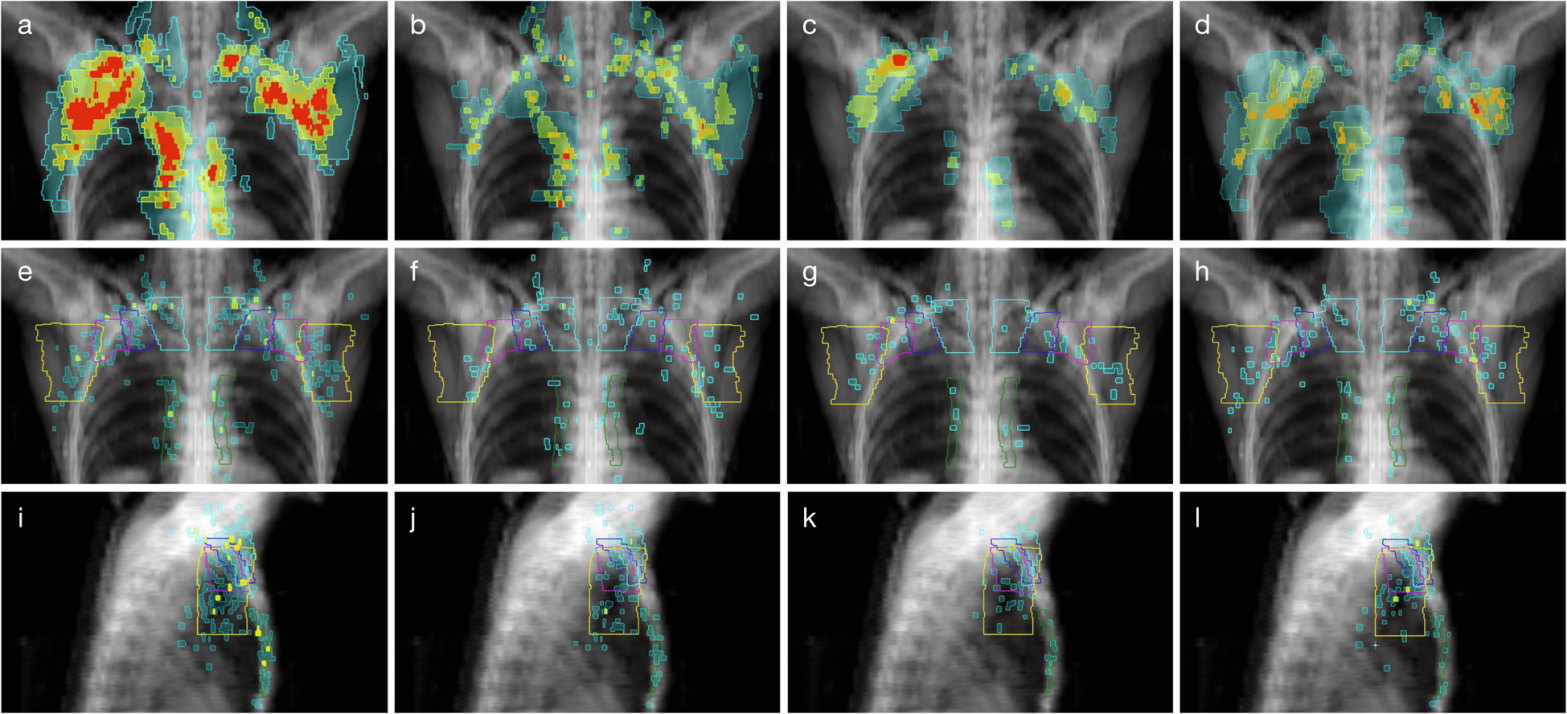
Anterior and lateral views of RNRs and RNR epicentres. Anterior view of contoured RNRs (**a**-**d**)*: **a** all patients, **b** nodal RT, **c** tangents **d** no RT; anterior view of RNR epicentres (**e**-**h**)*^#^: **e** all patients, **f** nodal RT, **g** tangents, **h** no RT; lateral view of RNR epicentres (**i**-**l**)*^#^: **i** all patients, **j** nodal RT, **k** tangents, **l** no RT. *Colour scheme of overlap of contoured nodes: Blue = one node, yellow = overlap of two nodes, Orange = overlap of three nodes, Red = overlap of four nodes. ^#^Colour scheme of RTOG-CTVs: Cyan = Supraclavicular; Yellow = Axilla level I; Magenta = Axilla level II; Blue = Axilla level III; Green = IMC

Figure 2 shows the location of all contoured nodes and epicentres by adjuvant treatment. An anatomic atlas of the 226 RNR, coded by initial treatment, is available in the supplemental material. The sites of RNR were: axilla level I (*n* = 64, 28%), followed by axilla level II (*n* = 37, 16%), SCF (anterior neck, *n* = 35, 15%), axilla level II (*n* = 35, 15%), IMN region (*n* = 34, 15%) and RADCOMP Posterior Neck (*n* = 21, 9%). Table 3 summarises the location of epicentres that were marginal/outside the RTOG and ESTRO-CTVs. For the RTOG SCF RNRs, 48% of epicentres were inside the SCF-CTV. Among the outside/marginal SCF epicentres, 18 were located posterior to the SCF-CTV in the Posterior Neck volume of the RADCOMP atlas. For the axillary RNRs, 91% of level I, 68% of level II, and 80% of level III epicentres were inside the axillary CTVs. Among the outside/marginal axillary epicentres, 12 were located caudal to the RTOG level II CTV. For the IMN RNRs, 68% of epicentres were inside the IMN CTV. Among the outside/marginal IMN epicentres, 7 were medial to the RTOG-CTV. Relapses in the interpectoral nodes were coded as outside the RTOG-CTV. Seventy-five percent (168/226) of RNRs were within the ESTRO-CTVs. For the ESTRO-SCF RNRs, 39% were inside the SCF CTV. Among the outside/marginal SCF epicentres, 18 were in the Posterior Neck volume of the RADCOMP atlas and 5 were cranial to the ESTRO-SCF CTV, but within the RTOG SCF CTV. For the axillary RNRs, 87% of level I, 78% of level II, and 85% of level III epicentres were inside the axillary CTVs. Eleven RNRs were within the interpectoral CTV. Among the outside/marginal axillary epicentres, 3 were posterior to the ESTRO level I CTV, but within the RTOG level I CTV. For the IMN RNRs, 85% of epicentres were inside the IMN CTV. Among the outside/marginal IMN epicentres, 3 were medial to the ESTRO-CTV.

**Table 3:** Locations of epicentres that were marginal or outside of RTOG and ESTRO Nodal CTVs

**RTOG CTVs**		**Classification of RNRs** **(Percentage for RTOG CTV)**			**Direction of Marginal or Outside RNR compared to RTOG CTVs** **Number (Percentage of all 68 Marginal and Outside RNRs)**					
	**RNR** ***n = 226***	**Outside** ***n = 60***	**Marginal** ***n = 8***	**Inside** ***n = 158***	**Cranial**	**Caudal**	**Anterior**	**Posterior**	**Lateral**	**Medial**
SCF	56	28 (50%)	1 (2%)	27 (48%)	5 (7%)			21 (31%)*	3 (4%)	
Axilla level I	64	6 (9%)	0	58 (91%)	1 (1%)	1 (1%)	1 (1%)	1 (1%)	2 (3%)	
Axilla level II	37	11 (30%)	1 (3%)	25 (68%)	3 (4%)	12 (18%)†	2 (3%)			
Axilla level III	35	9 (17%)	1 (3%)	25 (80%)	3 (4%)	3 (4%)		1(1%)	1 (1%)	2 (3%)
IMC	34	6 (18%)	5 (15%)	23 (68%)		2 (3%)			2 (3%)	7 (10%)
* RADCOMP Posterior Neck	21	3 (14%)°	0	18 (86%)	3 (100%)					
**ESTRO-CTVs**		**Classification of RNRs** **(Percentage for ESTRO-CTV)**			**Direction of Marginal or Outside RNR compared to ESTRO-CTVs** **Number (Percentage of all 61 Marginal and Outside RNRs)**					
	**RNR** ***n*** **= 226**	**Outside** ***n*** **= 57**	**Marginal** ***n*** **= 4**	**Inside** ***n*** **= 165**	**Cranial**	**Caudal**	**Anterior**	**Posterior**	**Lateral**	**Medial**
SCF	56	33 (59%)	1 (2%)	22 (39%)	9 (15%)			21 (34%)*	3 (5%)	1 (2%)
Axilla Level I	64	8 (13%)	0	56 (87%)	1 (1%)	1 (1%)	1 (1%)	4 (7%)	1 (1%)	
Axilla Level II	27	6 (22%)	0	21 (78%)	3 (5%)	1 (1%)		2 (3%)		
Interpectoral	11	0	0	11 (100%)						
Axilla Level III	34	7 (12%)	1 (3%)	26 (85%)	3 (5%)	3 (5%)				2 (3%)
IMC	34	3 (9%)	2 (6%)	29 (85%)		2 (3%)				3 (3%)
* RADCOMP Posterior Neck	21	3 (14%)°	0	18 (86%)	3 (100%)					

*Abbreviation*: *RNR* Regional nodal recurrence

### Factors related to CTV coverage

To determine which node-positive patients need more extensive nodal coverage, associations between baseline variables and RNR epicentres being marginal/outside of the ESTRO-CTV were analysed using univariable logistic regression. We report our analysis of geographic misses cranial to the SCF-CTV or in the posterior neck in Table 4. There was no significant association between geographic miss for the baseline variables: grade, LVI, use of RNI, type of relapse, or stage. However, the odds ratio of having supra-ESTRO SCF-CTV or RADCOMP Posterior Neck RNRs was elevated: 3.3 (0.4–29.0, *p* = 0.28) for patients with stage 3 vs stage 1–2 disease.

**Table 4:** Univariable logistic regression analysis of risk factors for regional nodal recurrences superior to the ESTRO supraclavicular CTV (CTV4) or in the RADCOMP Posterior Neck CTV in patients who were treated for node-positive breast cancer (*n* = 33)

Baseline Variable	Odds ratio	95% confidence interval	*p*-value
Lymphovascular invasion*			
Yes vs No	1.8	0.3–10.9	0.52
Grade*			
3 vs 1 & 2	0.6	0.1–3.0	0.56
Stage*			
III vs I & II	3.3	0.4–29.0	0.28
**Adjuvant regional nodal radiotherapy**			
Yes vs No	0.7	0.1–3.5	0.63
**Type of relapse**			
Metastatic & RNR vs RNR only	0.6	0.1–3.0	0.56

*Abbreviation*: *RNR* Regional nodal recurrence. *Unknowns removed before statistical analyses

## Discussion

In this study, we used PET/CT to create a comprehensive atlas of RNR after curative treatment. Strengths of our study include exclusive use of PET/CT and our well-defined cohort of relapse-only patients who were imaged before any salvage treatment. Our study showed that SCF RNR coverage by the SCF-CTVs was poor: only 48% for the RTOG atlas and only 39% for the ESTRO atlas. Almost one-third of the supraclavicular RNR outside of the RTOG and ESTRO SCF-CTVs were located within the RADCOMP Posterior Neck volume [25]. The ESTRO IMC-CTV is 5 mm wider than the RTOG IMC-CTV, the ESTRO axilla Level II is more generous inferiorly than the RTOG axilla level II CTV and the ESTRO interpectoral CTV covered RNR missed by the RTOG-CTV. We observed that these differences were responsible for the per-patient increase in coverage of all RNRs from 38% of patients by the RTOG-CTV to 57% of patients with the ESTRO-CTV. Addition of the RADCOMP Posterior Neck volume increased coverage of all RNRs to 48% for the RTOG-CTV and to 70% for the ESTRO-CTV.

There are four studies that have mapped the location of RNRs in relation to the RTOG nodal atlas using a mixture of patients at diagnosis and at relapse, imaged with CT or PET/CT [13–16]. Two studies focused on SCF coverage: Jing et al demonstrated that the RTOG SCF CTV covered 62.6% of SCF epicentres across all patients and all SCF epicentres in 25.5% of patients, whilst Brown et al showed that the RTOG SCF CTV covered 59% of SCF epicentres [13, 15]. In our study, the RTOG SCF CTV covered only 48% (27/56) of epicentres, possibly because our cohort was entirely relapsed patients. A third study by Jethwa et al evaluated IMN epicentres and reported 53% overall coverage with 19% of IMN nodes located medial to the RTOG IMN CTV [16]. In our study, 18% (6/34) of the IMN marginal/outside epicentres were medial to the RTOG IMN CTV, which includes only the vessels. Jethwa et al suggested adding a 4 mm margin transversely to the RTOG IMN CTV. The caudal border of the RTOG level II CTV was highlighted as a high-risk area by Gentile et al, who showed that 80% of uncovered level II RNR were located caudal to the RTOG level II CTV [14]. Similarly, 71% (12/17) of our level II ‘marginal/outside’ epicentres were located caudally.

Borm et al studied patients with both primary and recurrent disease by contouring the nodes with FDG uptake on CT/PET and deformably mapping them onto the CT of a template patient with the atlases contoured [19]. They excluded lymph nodes with volume > 20 cm^3^, corresponding to an equivalent spherical recurrence of 3.3 cm diameter. In the 197 patients with 410 RNR (mean 2.1 RNR/patient), 27.3% of nodes were 95% covered, 52.2% of nodes had 5–95% coverage and 20.5% of nodes were < 5% covered by the RTOG-CTV. They also noted that a higher proportion of FDG-avid SCF and IMC nodes were observed in the relapse setting than for newly staged patients, for example, 19.5% of nodes were located in the SCF in patients with recurrent breast cancer, which was 5 times higher compared to patients with primary breast cancer. This is in keeping with our results: we found that 25% of RNRs were in the SCF.

DeSelm et al studied 243 RNR in 153 patients (mean 1.6 RNR/patient) using an unreported mix of CT and PET imaging by manually transferring the location of RNR epicentres onto a template patient. They found that baseline LVI was associated with IMN RNR and that Grade 3 disease was associated with IMN and SCV RNR. The RTOG-CTV encompassed 82% of RNR. In addition, the majority 67% (30/45) of out-field RNR were located in the lateral and posterior SCF region. They made an important observation that estrogen receptor-negative status, lymphovascular invasion, and grade 3 disease independently predicted SCV and IMN recurrence [20].

Chang et al studied 337 treatment-naive local and regional recurrences in 234 patients imaged with an unknown mix of CT and PET imaging [18]. There were 237 RNR in 162 patients (mean 1.5 RNR/patient). They reported differences in coverage between the RTOG-CTV and the ESTRO-CTV in relation to the ESTRO-CTV. The ESTRO-CTV covered 100% of Axilla Level 1, 94% of Axilla Level II, 95% of Axilla Level III and 100% of IMC RNR. However, the coverage was not as good for the supraclavicular region: the ESTRO-SCF-CTV covered only 71% of SCF RNRs and, while the RTOG-SCF-CTV covered an additional 9% of SCF RNRs superior to the ESTRO-SCF-CTV, 20% were outside both CTVs. We found that a similar 9% (5/56) of SCF recurrences occurred superior to the ESTRO-SCF-CTV, but inside the RTOG-SCF-CTV. In our study, the RTOG-SCF-CTV covered only 48% and the ESTRO-SCF-CTV covered only 39% of SCF-RNR. This difference may be due to our exclusive use of PET/CT, which is superior to CT for detection of lymph node metastasis [24, 29, 30]. There may also have been a selection bias in the use of PET/CT in our cohort, which is suggested by our patients having a median age of 49 years and our mean RNR/patient being more than double that in their study. Almahariq et al. studied the location of biopsied axillary level I lymph nodes at initial diagnosis in relation to the RTOG, ESTRO and RADCOMP atlases. The level 1 axillary CTVs covered 54–66% of the biopsied lymph nodes, because many were lateral and inferior to the axillary CTVs below the level of the clavicle. However, when the breast CTV was considered, 94% of the lymph nodes were covered by the RTOG atlas [21].

In our study, we confirmed that RNRs outside the RTOG and ESTRO-CTVs were common. Other studies have shown that the lateral and posterior aspects of the SCF are not well covered [13, 15, 17, 18, 20]. Previous suggestions to modify the SCF-CTVs have included; extending to the lateral-most extent of the scalene muscle, having a more generous coverage of the posterior SCF [13], and modifying the SCF to its natural anatomic barriers (encompassing the medial edge of the trapezius muscle, lateral edge of the thyroid gland, posterior edge of the SCM muscle and ventral edge of the trapezius muscle), which has been shown to encompass 96.1% of all nodes [15]. The “Posterior Neck” volume of the RADCOMP atlas and the “Lateral SCV” and “Posterior SCV” volumes in DeSelm et al’s manuscript supplement both provide good descriptions of this important region of geographic miss.

Our study does have several limitations. Importantly, we cannot know whether we were studying RNR in lymph nodes that did not contain microscopic disease at presentation because the anatomic extent of nodal disease increased between the time of curative treatment and RNR detection. Hence, our reported extent of RNRs may be an overestimate of the extent of microscopic disease in typical patients at the time of treatment. In addition, our cohort included a number of patients initially treated in the 1990’s when cytotoxic chemotherapy was less effective in an era when HER-2 testing and targeted therapies were not available. It is possible that the use of modern adjuvant therapies may have modified patterns of RNR. Also, although 1071 breast cancer patients underwent PET/CT in our study period, only 69 patients met inclusion criteria. Our sample size was further divided into three subsets that received different initial locoregional management, limiting our ability to study how initial treatments impacted relapse patterns.

In this study, RNRs were contoured using the highly reproducible PET Edge™ tool in the MIM software, which was calibrated so that the PET-generated contours matched the visible masses on CT. An alternative approach would have been to contour the FDG-avid RNRs using the CT scan alone. However, our approach was taken for a number of reasons. Firstly, we found that many axillary relapses were surrounded by surgical clips, meaning that the CT images were degraded by artefact, which would have led to a degree of uncertainty when contouring on CT alone. It is not unusual for a normal lymph node to measure 2 cm in the long axis, but to have eccentric FDG-uptake, for example, at one pole. Secondly, contouring of the entire lymph node, most of which is normal, on the CT scan would lead to a misrepresentation of relapse location. Trying to contour only part of the lymph node would lead to uncertainty about the edges of the relapse within the cortex of the lymph nodes because the density of normal lymph node cortex and metastatic disease are similar on CT. However, we did review the relationship between FDG-uptake and CT masses for every RNR and discovered that a small number of patients had moved between the attenuation-correction CT and PET acquisition. For these cases, the RNRs were recontoured exclusively on the CT/PET thus improving the accuracy of our results. Furthermore, we decided to report on epicentre location as opposed to the location of the entire RNR contour. The majority of studies of regional nodal relapses after breast cancer treatment have also analysed the epicentres of contoured RNRs, but we do appreciate that other excellent studies have instead analysed the full extent of the contoured RNRs (Table 1). It is our personal observation that RNRs are usually spherical or ellipsoid in shape, in keeping with previous reports [27, 28]. We therefore believe that the centroid of a focus of FDG uptake or a mass on CT is an imperfect, but adequate, surrogate for the original tumour nidus. However, for completeness, in Fig. 2a, we do illustrate the full extent of the contoured RNRs in our study.

With advanced radiotherapy techniques, such as IMRT and proton beam therapy being used for RNI, precise CTV target definition is important to ensure accurate dose delivery. Kowalski et al. published a study including 389 RNRs in 102 patients (3.8 RNR/patient) [22]. They compared the dosimetric coverage of FDG-avid lymph node metastases for plans created with the RTOG, ESTRO and RADCOMP atlases using 3D conformal, volumetric-modulated arc therapy (VMAT) and pencil-beam scanning proton therapy (PBSPT). Reassuringly, they found that 97% of RNR were covered by a conventional 3D conformal plan of partial wide tangents and APPA supraclavicular and axillary fields. With dose distributions optimized for each of the three atlases, the RNR coverage by VMAT plans was 89% for ESTRO, 93% for RTOG and 98% for RADCOMP and by PBSPT plans was 88% for ESTRO, 91% for RTOG and 96% for RADCOMP. Only the sophisticated plans generated using the RADCOMP atlas achieved superior coverage of RNRs to a conventional 3D conformal plan. Hence, the RADCOMP atlas seems to identify the volumes most at risk of RNR better than the RTOG and ESTRO atlases. However, larger irradiation volumes could lead to an increased risk of late toxicity, including carotid artery stenosis, lymphedema, cardiac injury or secondary malignancy [31–34], and the additional benefit would need to outweigh these risks. Perhaps only patients with high risk disease need radiotherapy planning to the RADCOMP nodal CTVs. Unfortunately, our analysis did not identify a baseline variable that would justify contouring a posterior neck volume when treatment planning node-positive patients. Larger studies with more power to analyse baseline characteristics in relation to RNR locations may be able to identify predictive variables.

There are two review articles about validation studies of breast cancer nodal CTVs. Gee et al. performed a comparison and systematic review of contouring consensus guidelines for breast cancer radiotherapy, including the RTOG, ESTRO and RADCOMP atlases [35]. They concluded that more generous CTVs may be recommended for patients with locally advanced disease. Loganadane et al. also compared the nodal target volumes of the RTOG, ESTRO and RADCOMP atlases [36]. They provided detailed visual and written details about the differences between the atlases and concluded that treatment planning for patients with locally advanced disease should use the RADCOMP atlas. Our study results are congruent with the opinions expressed in these review articles. We found that the RTOG atlas did not cover RNRs as well as the ESTRO atlas and that the ESTRO atlas did not cover RNRs as well as the RADCOMP atlas. We performed a logistic regression analysis to identify patients who were more likely to have RNRs superior to the ESTRO SCF and/or in the RADCOMP Posterior Neck. Although the odds ratio was not statistically significant in our study, stage III patients were more likely than stage II patients to have RNRs in these regions.

## Conclusions

Our supplemental atlas of RNRs highlights areas at high risk of nodal relapse after curative treatment. The RTOG and ESTRO breast cancer nodal CTVs do not fully cover all potential areas of RNR, but the ESTRO nodal CTVs provided full coverage of all RNR epicentres in more patients than the RTOG nodal CTVs. With addition of the RADCOMP Posterior Neck volume to the ESTRO-CTVs, 70% of patients had full coverage of all RNR epicentres. We recommend use of the RADCOMP atlas for Stage III patients.

## Supplementary information

**Additional file 1.**

## Data Availability

The datasets used and/or analysed during the current study are available from the corresponding author on reasonable request.
