# Supplementary material for: PET/CT of breast cancer regional nodal recurrences: an evaluation of contouring atlases
Source: Radiat Oncol. 2020 Jun 1;15:136. doi: 10.1186/s13014-020-01576-6 (PMC7268399; doi:10.1186/s13014-020-01576-6)
Supplement: Supplementary file 1 — Additional file 1. [file 13014_2020_1576_MOESM1_ESM.pdf]

BC CANCER
Provincial Health Services Authority

- L CTV1 EORTC
- L CTV2 EORTC
- L CTV3 EORTC
- L CTV4 EORTC
- L IMN EORTC
- L IP EORTC
- R Ax1 RTOG
- R Ax2 RTOG
- R Ax3 RTOG
- R SCF RTOG
- R IMC RTOG
- L PN RADCOMP
- R PN RADCOMP

- Regional nodal recurrence after regional nodal radiotherapy
- Regional nodal recurrence after tangent radiotherapy
- Regional nodal recurrence after no adjuvant radiotherapy

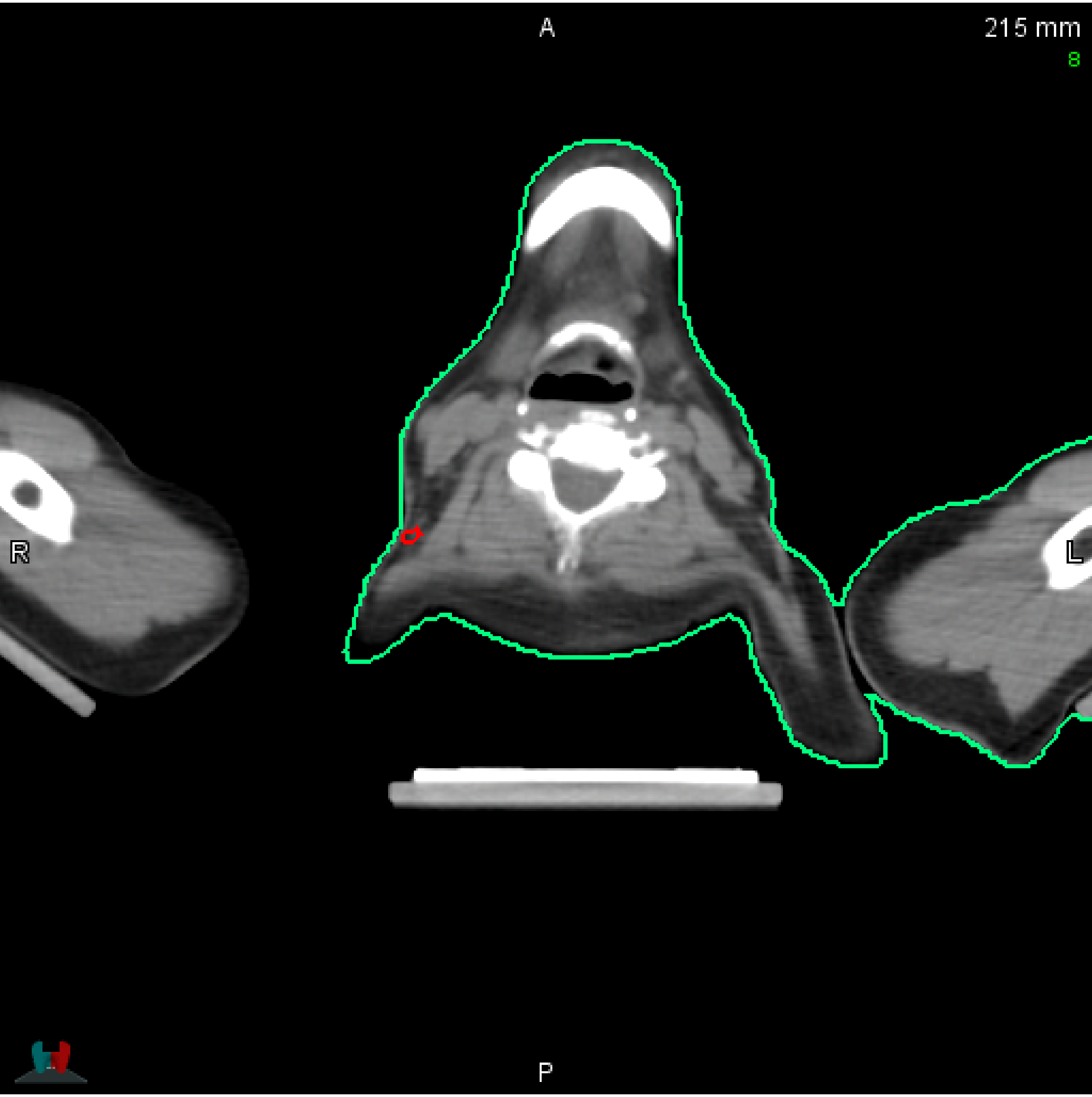
A
215 mm
8
R
L
P

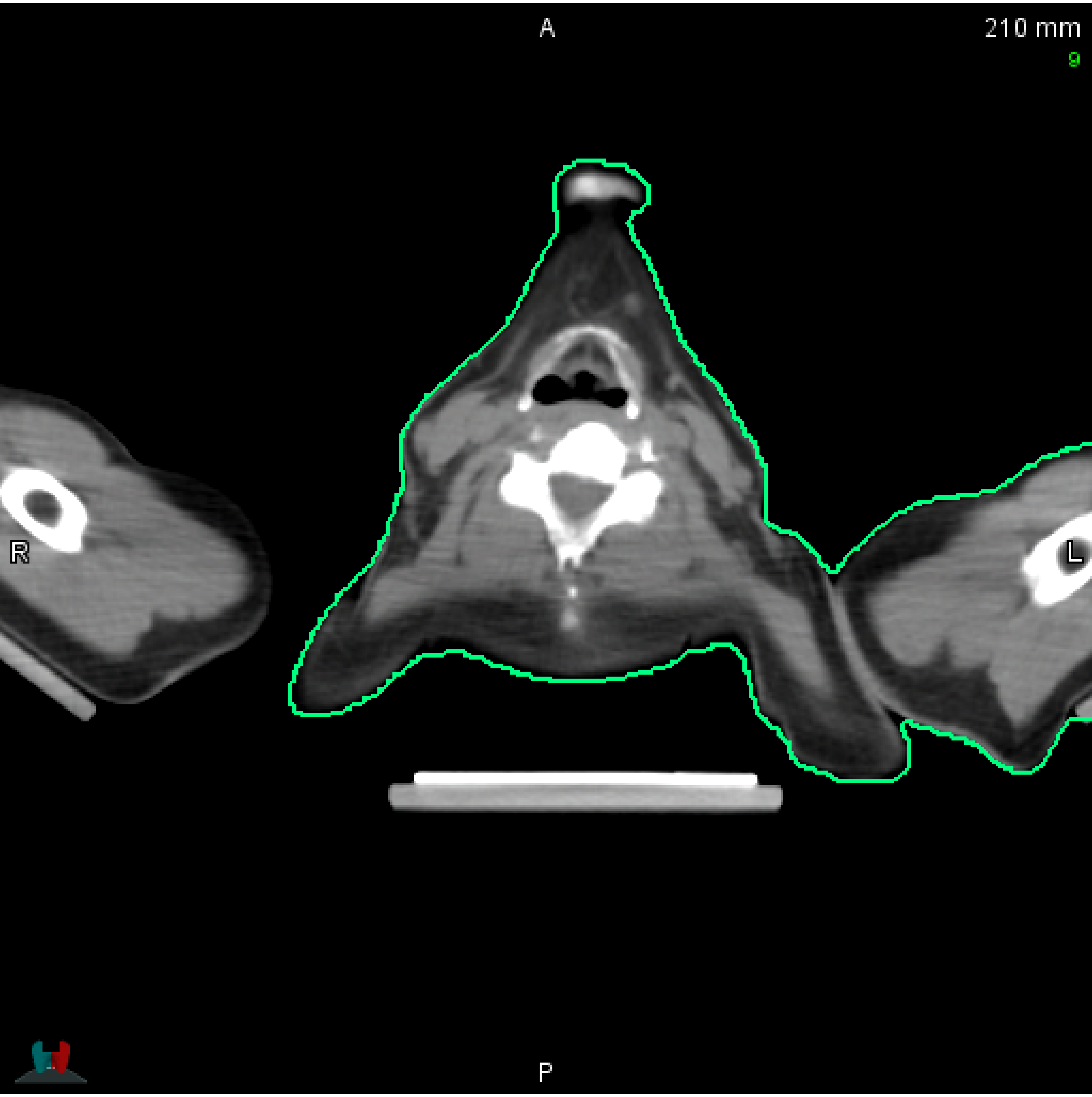
A
210 mm
9
R
L
P

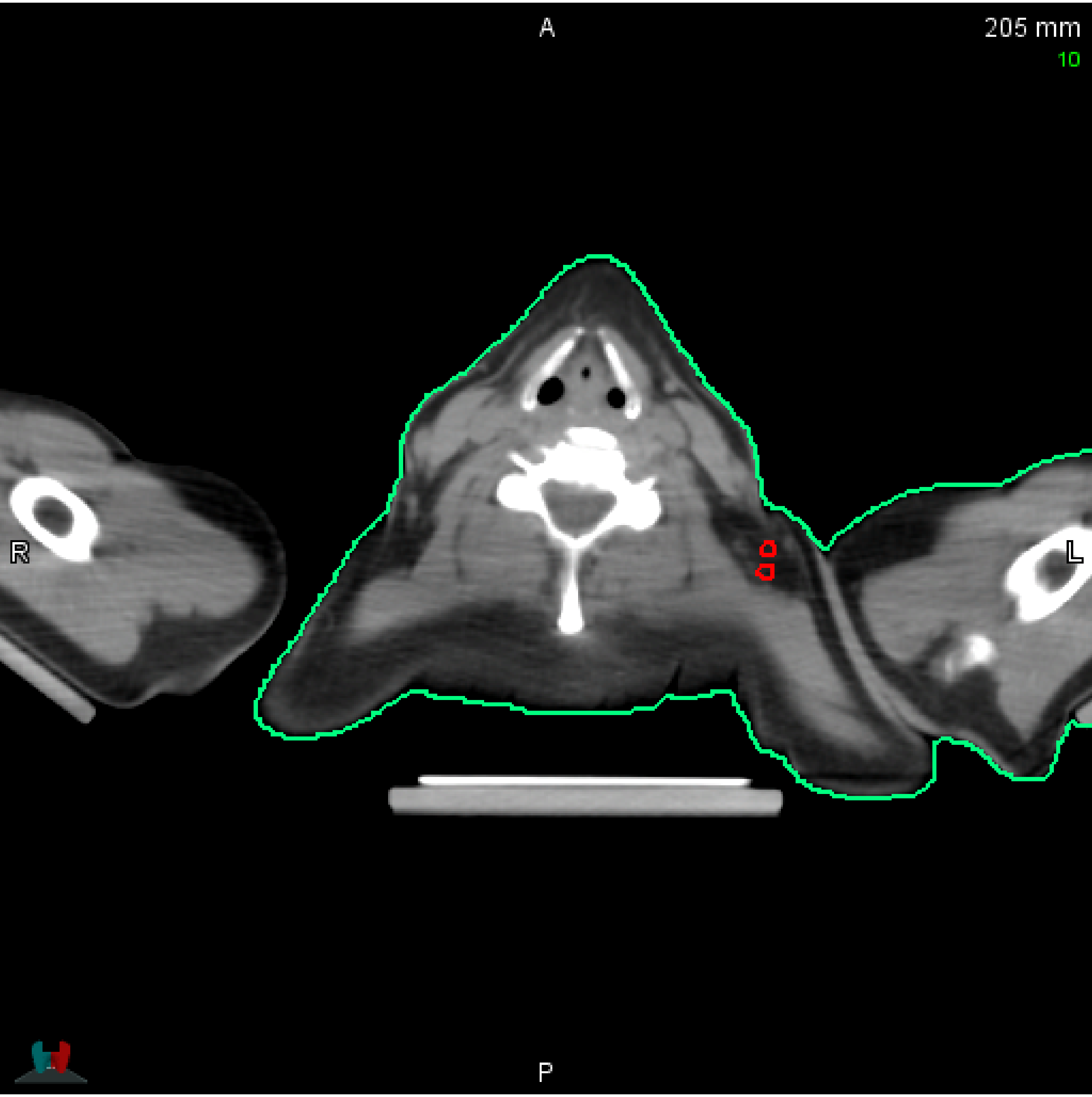
A
205 mm
10
R
L
P

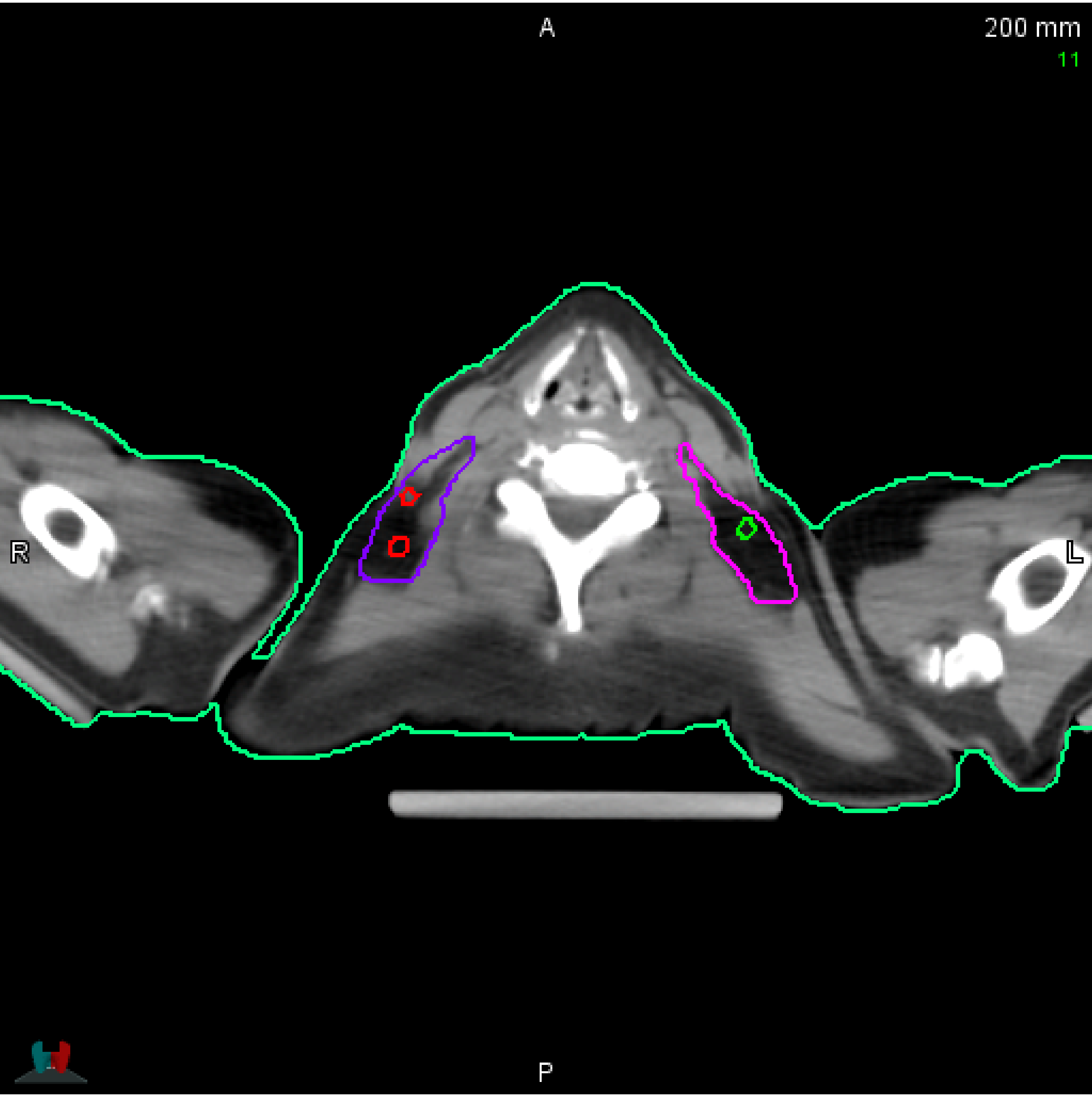
A
200 mm
11
R
L
P

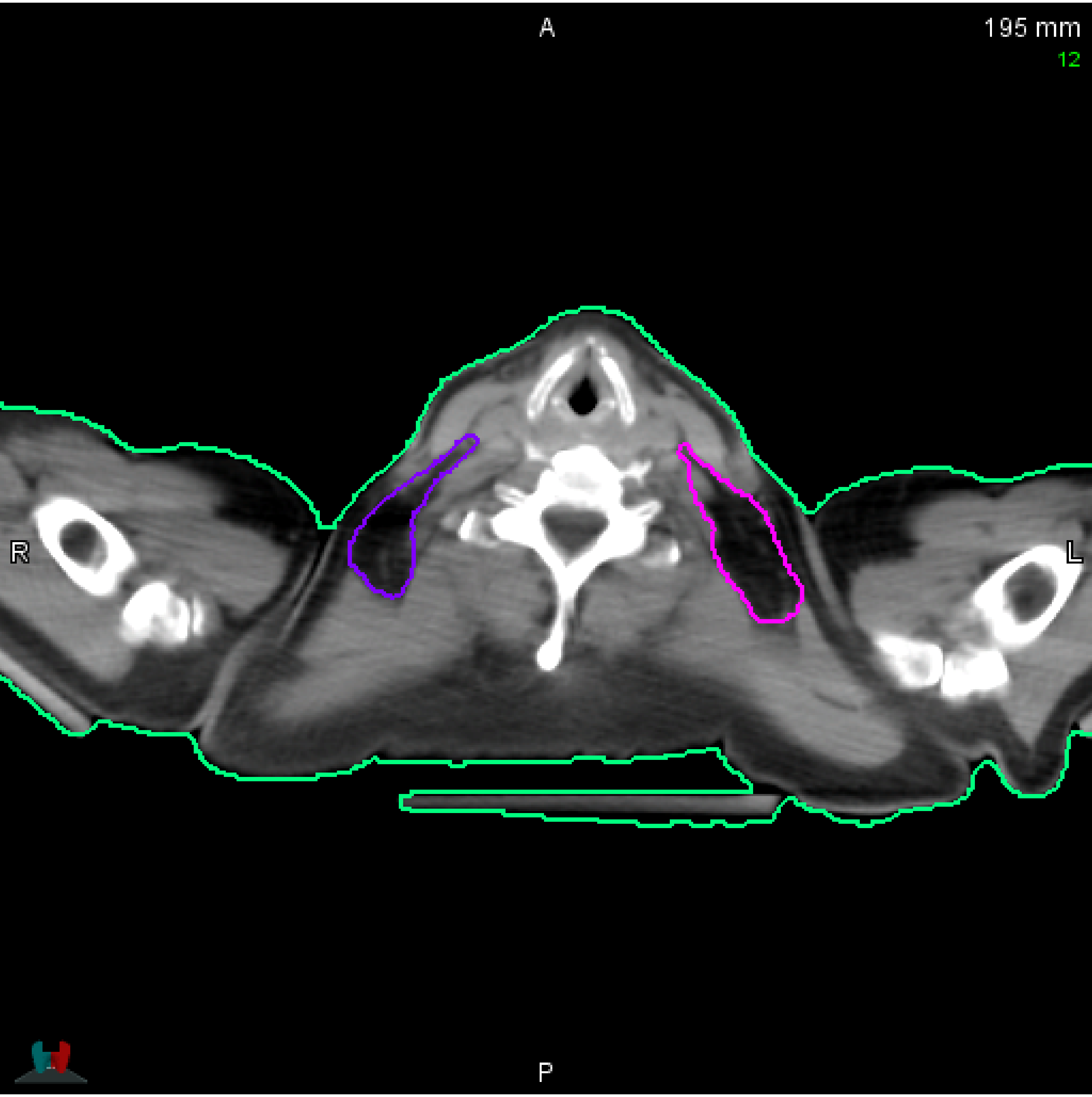

A
195 mm
12
R
L
P

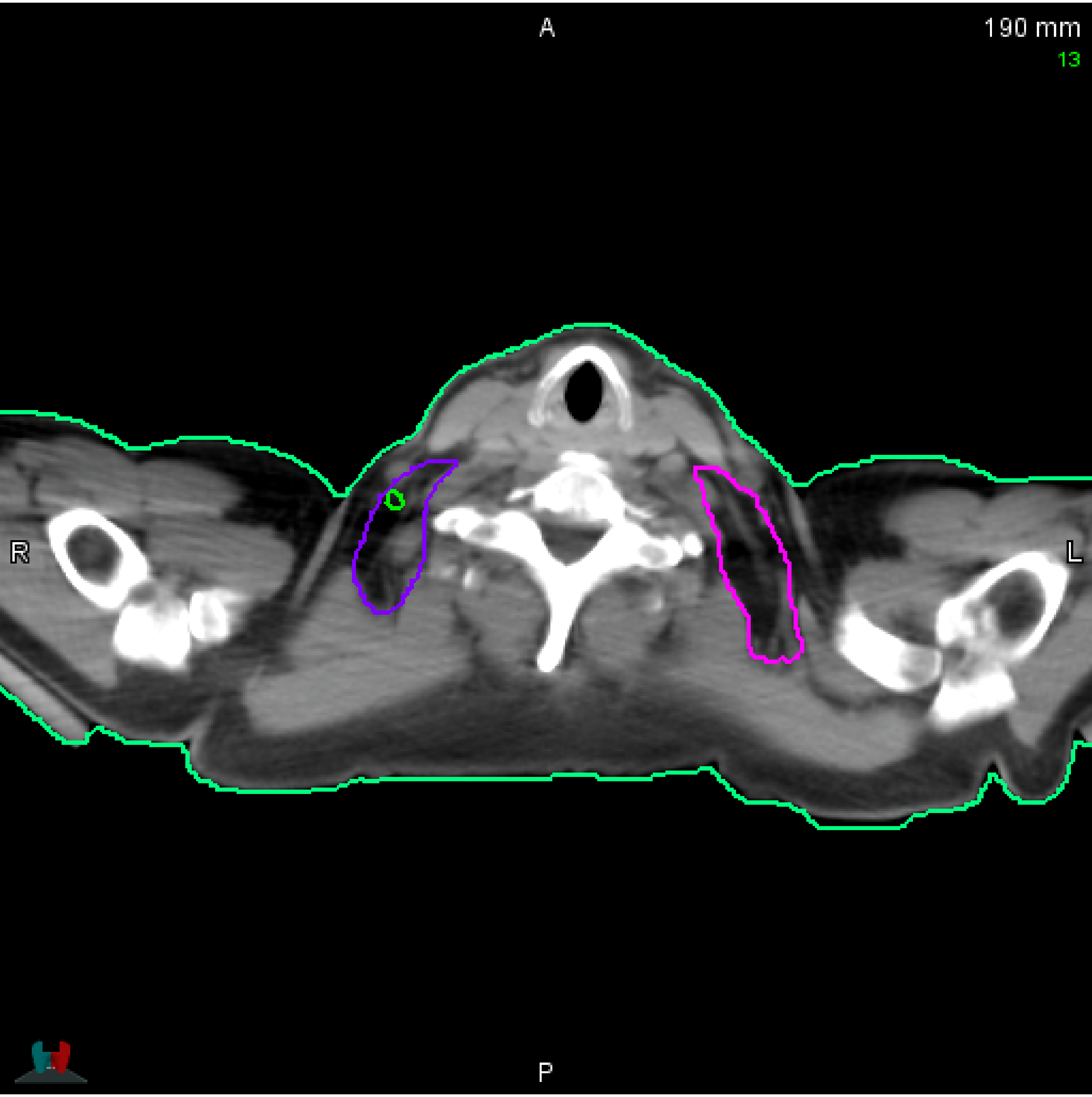
A
190 mm
13
R
L
P

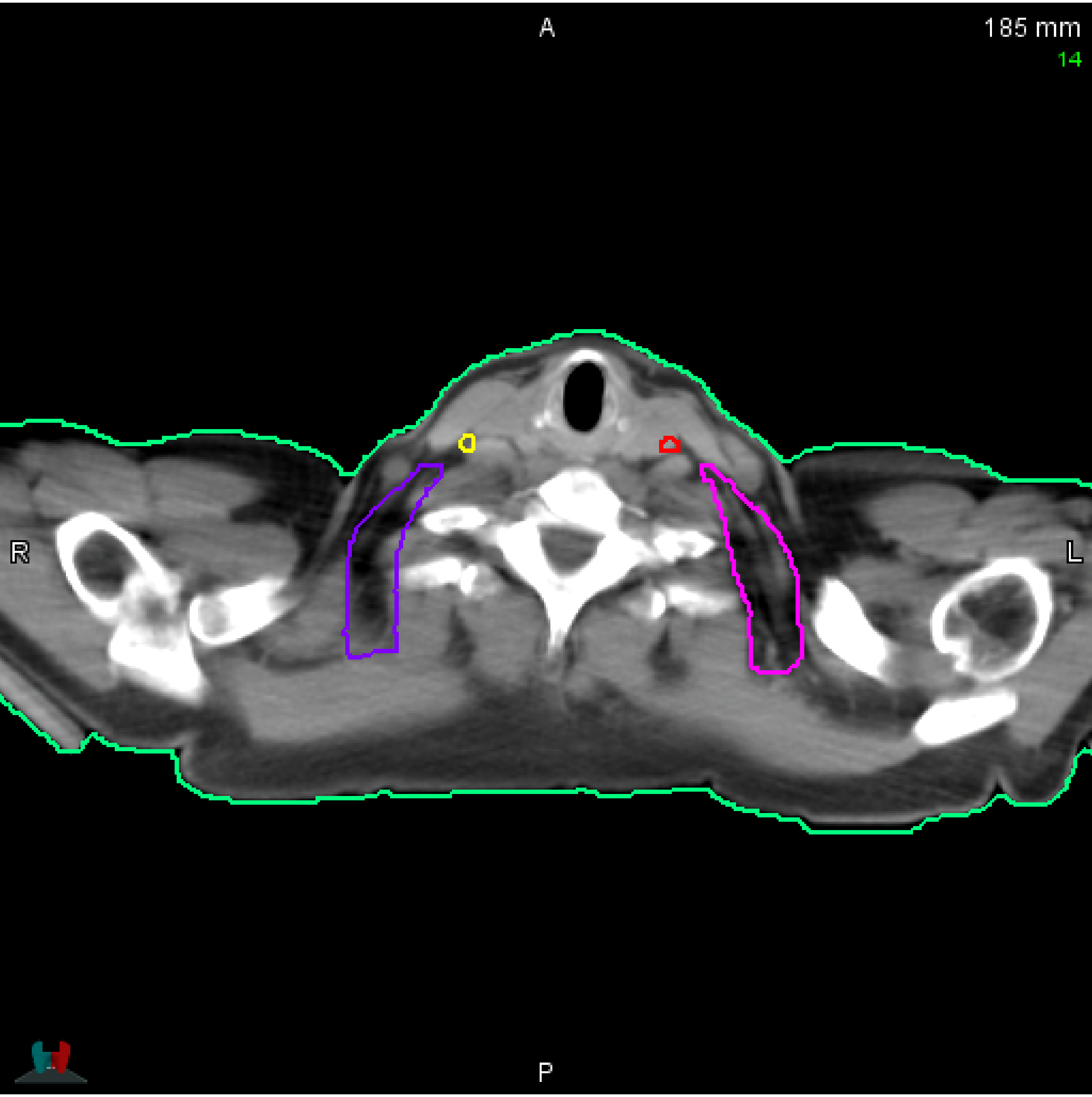
A
185 mm
14
R
L
P

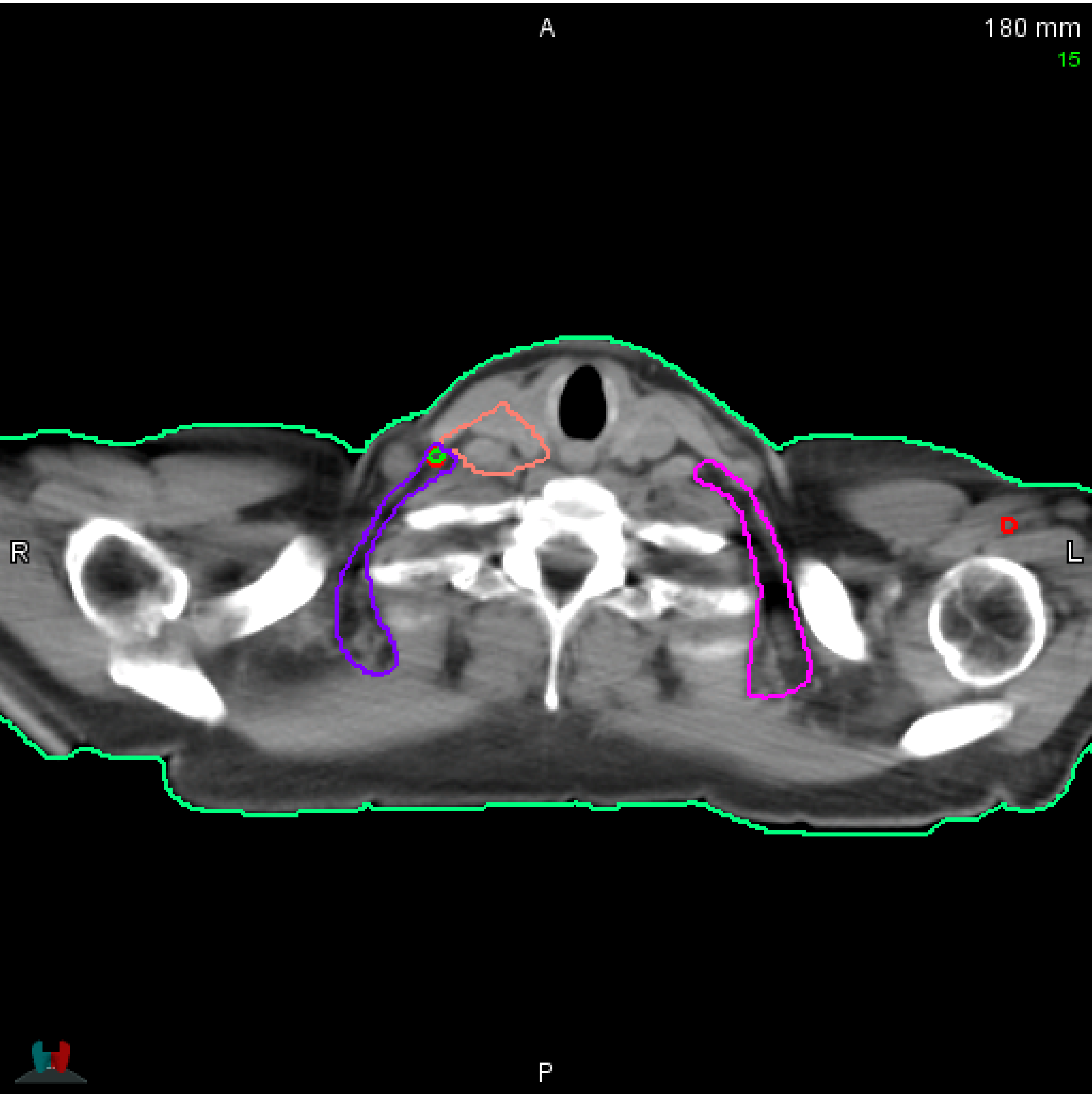
A
180 mm
15
R
L
D
P

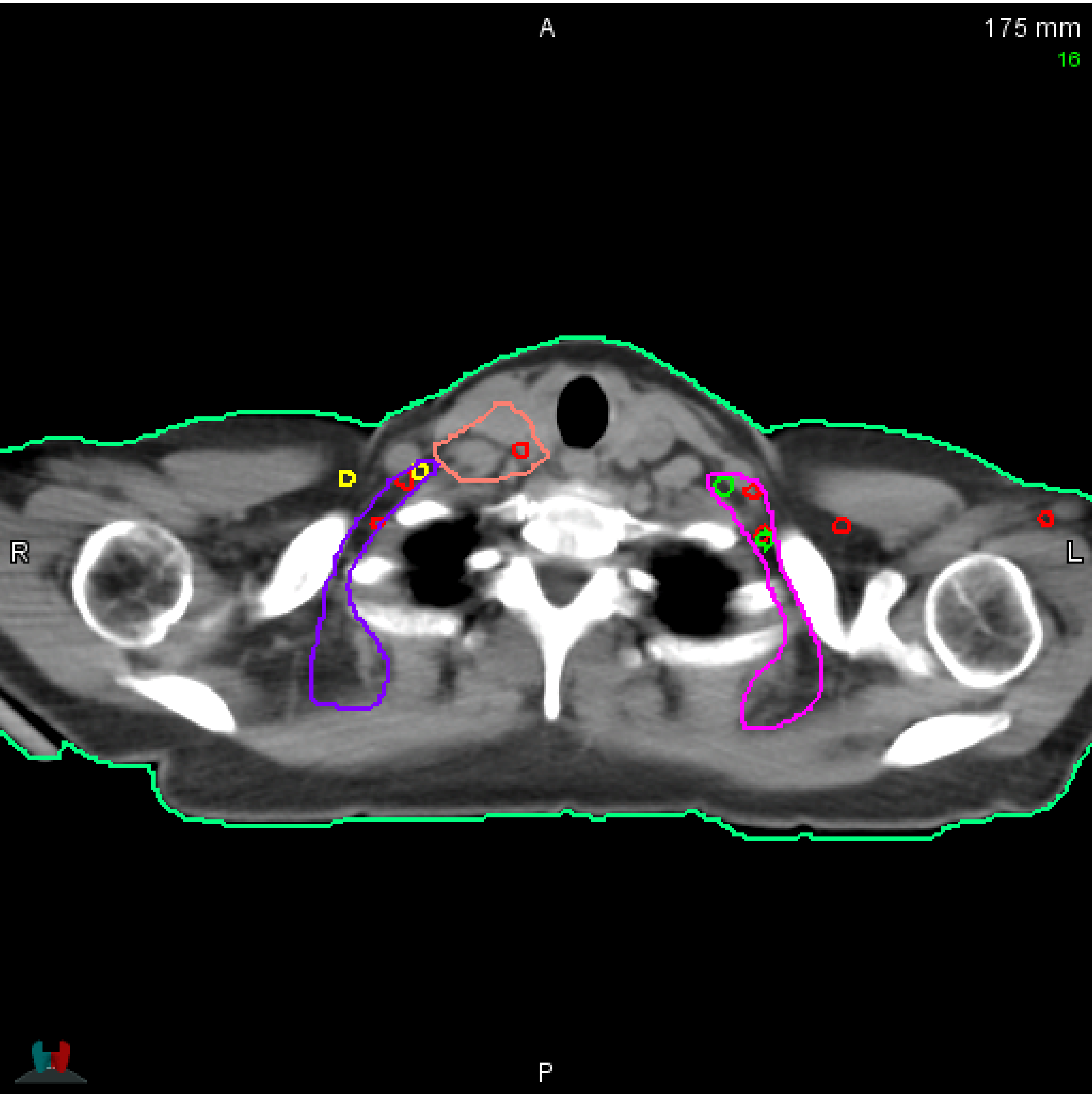
A
175 mm
16
R
L
P

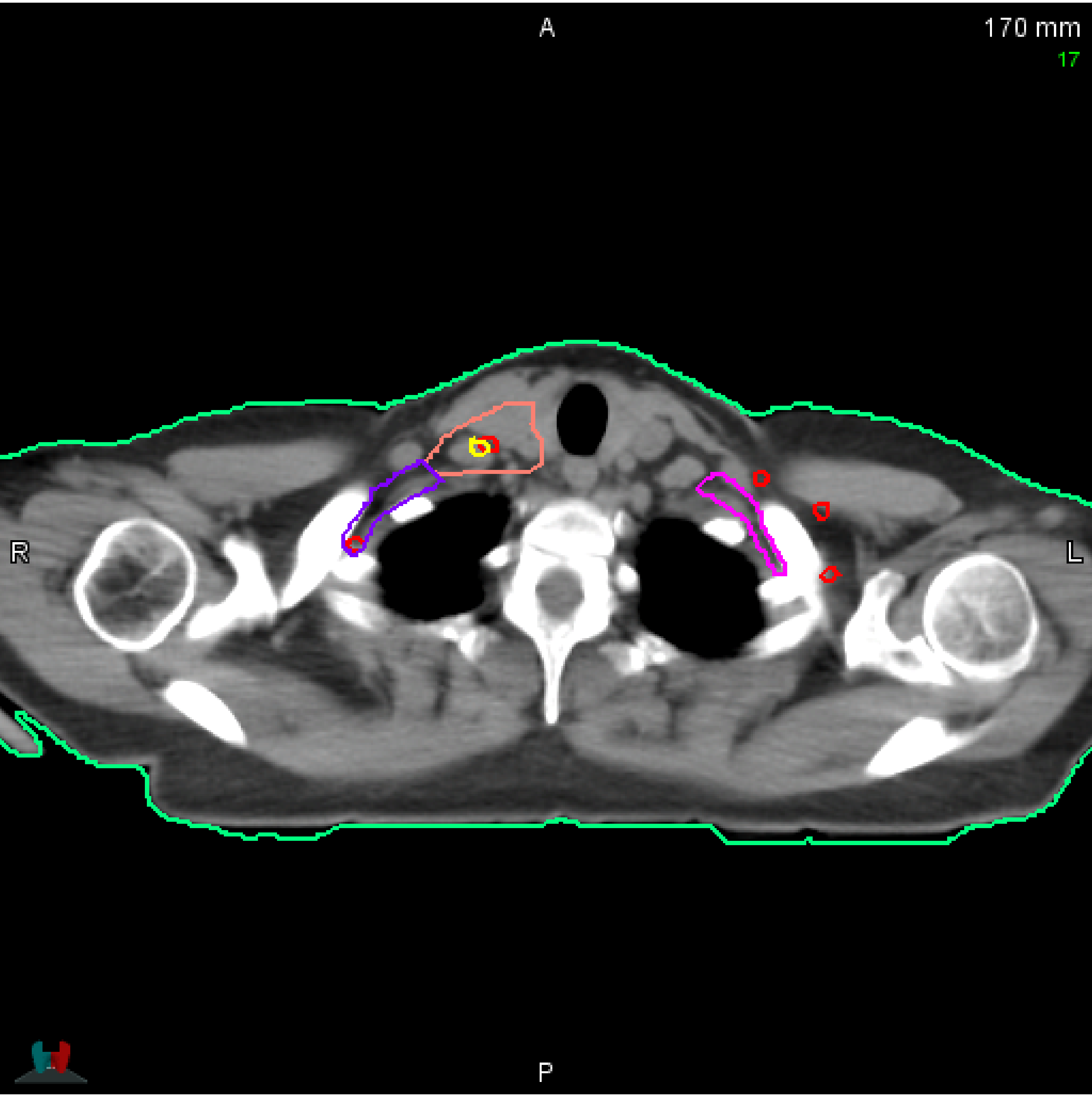
A
170 mm
17
R
L
P

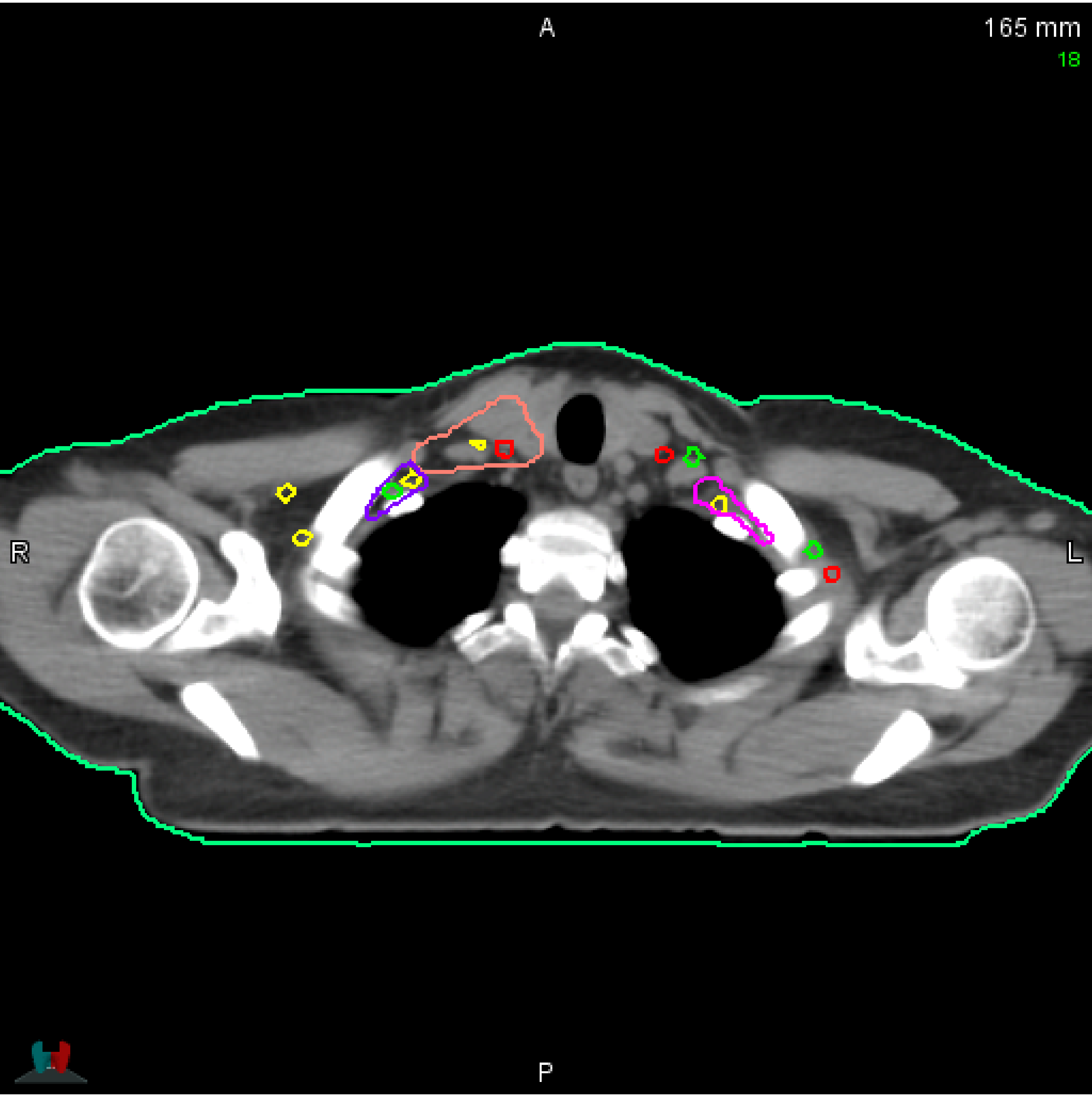

A
165 mm
18
R
L
P

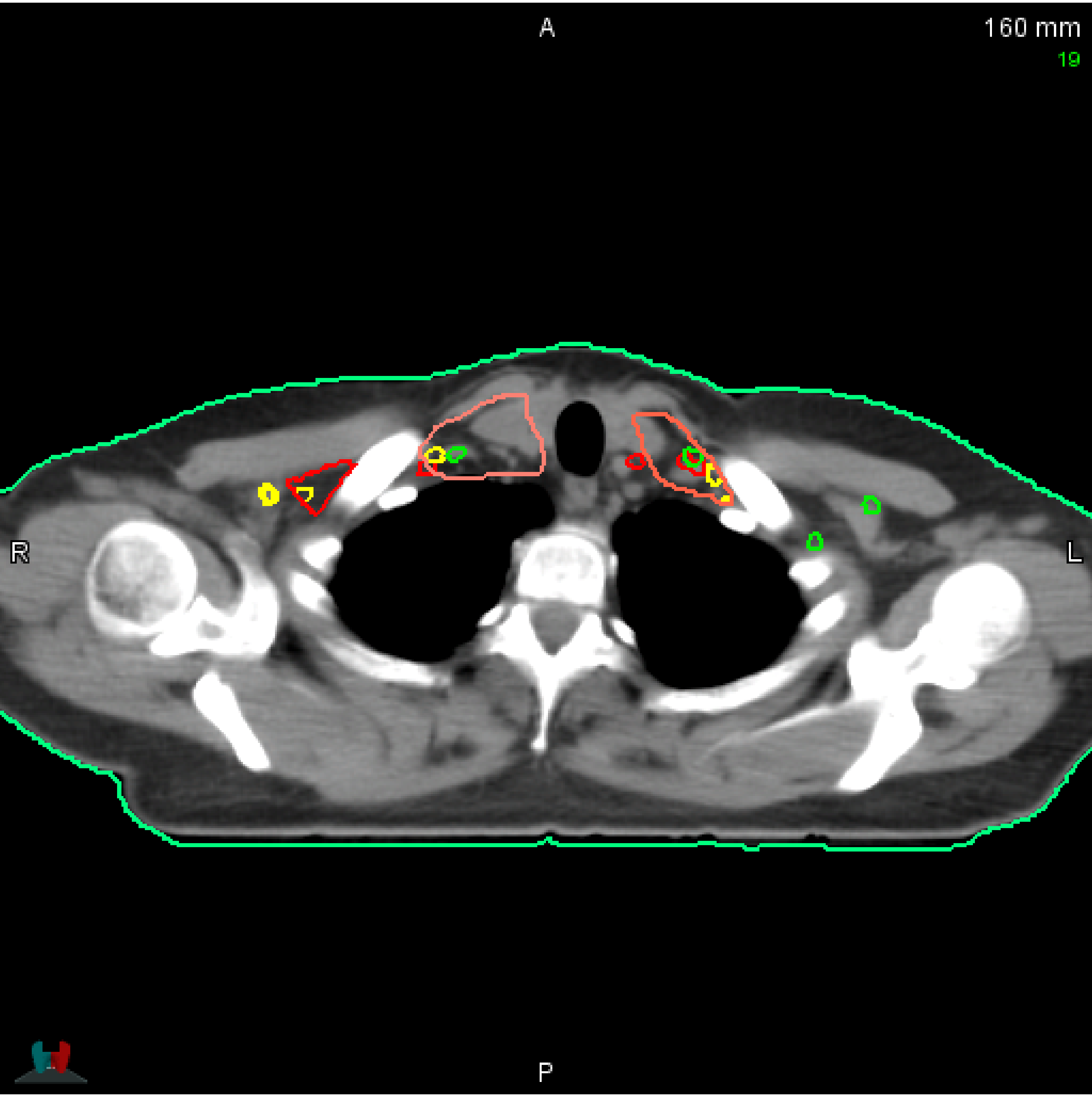
A
160 mm
19
R
L
P

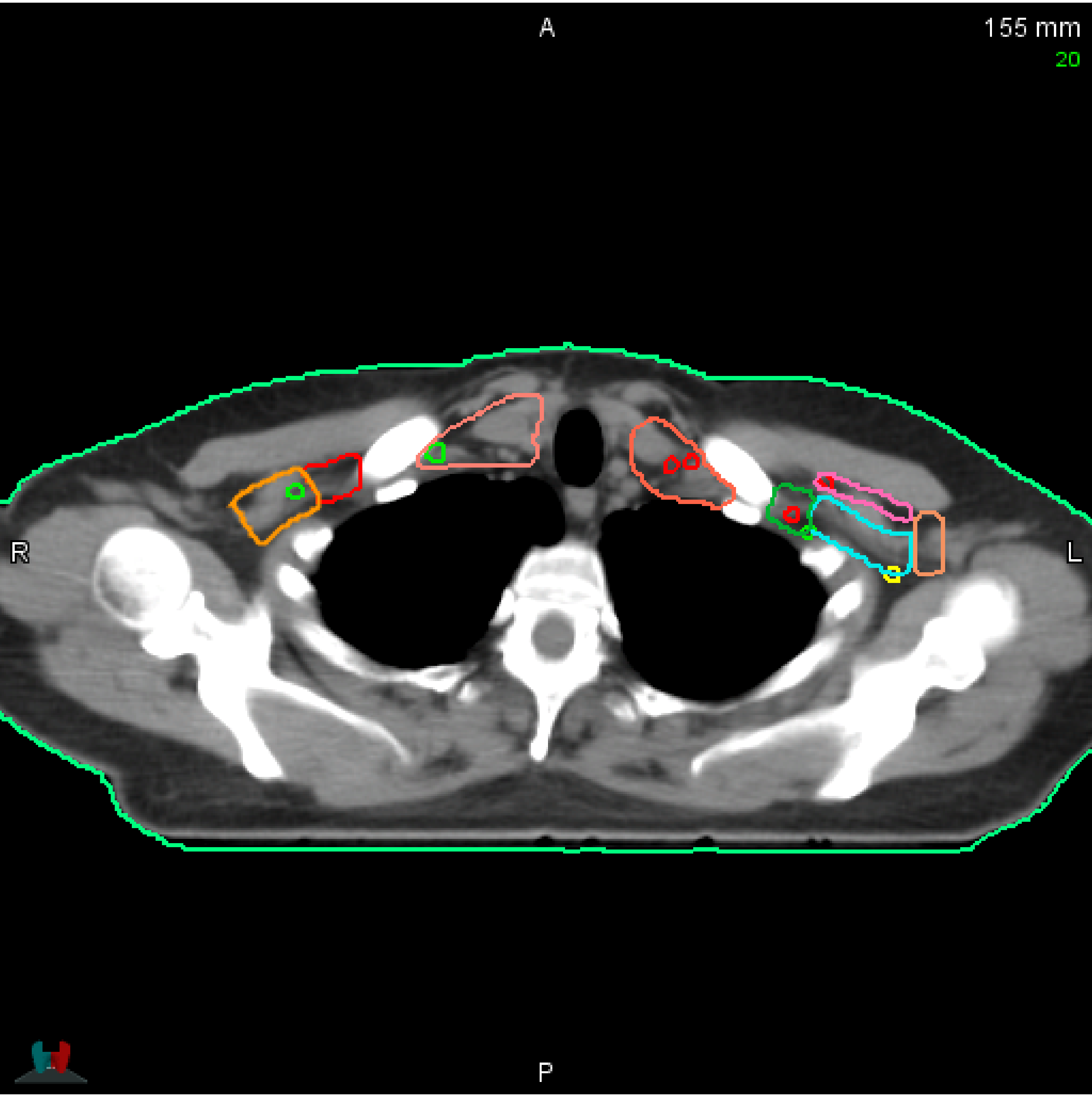
A
155 mm
20
R
L
P

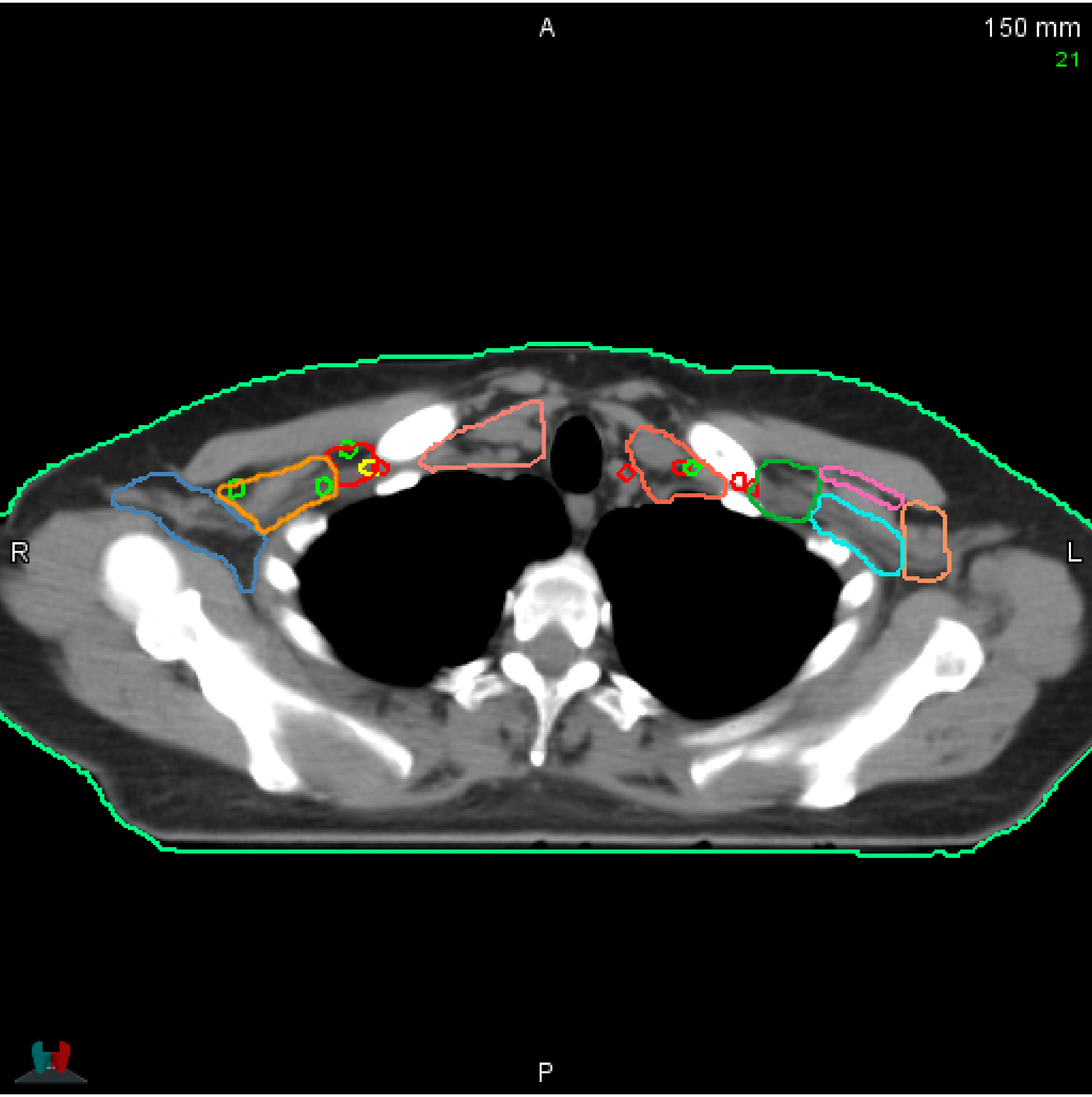
A
150 mm
21
R
L
P

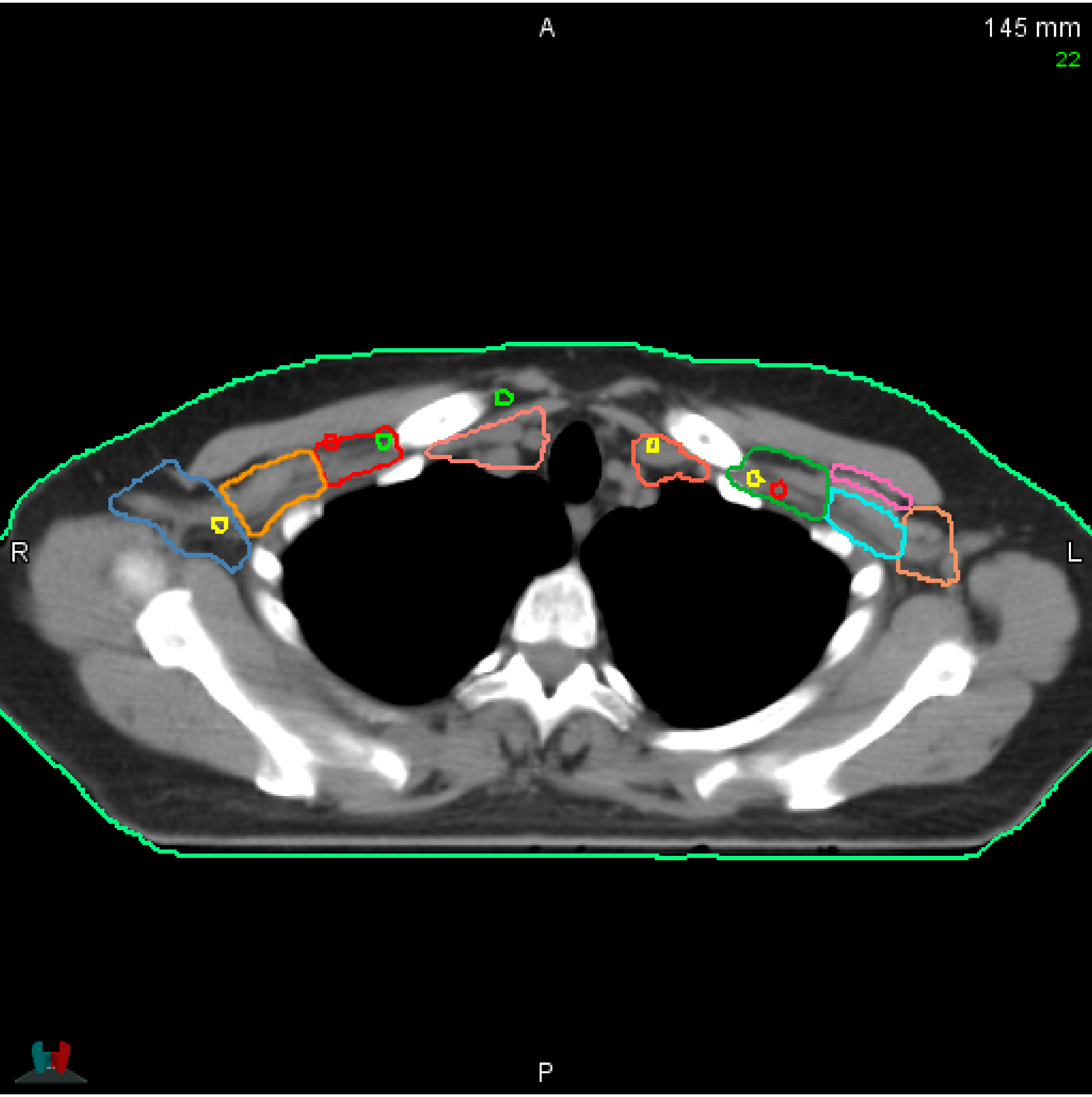
A
145 mm
22
R
L
P

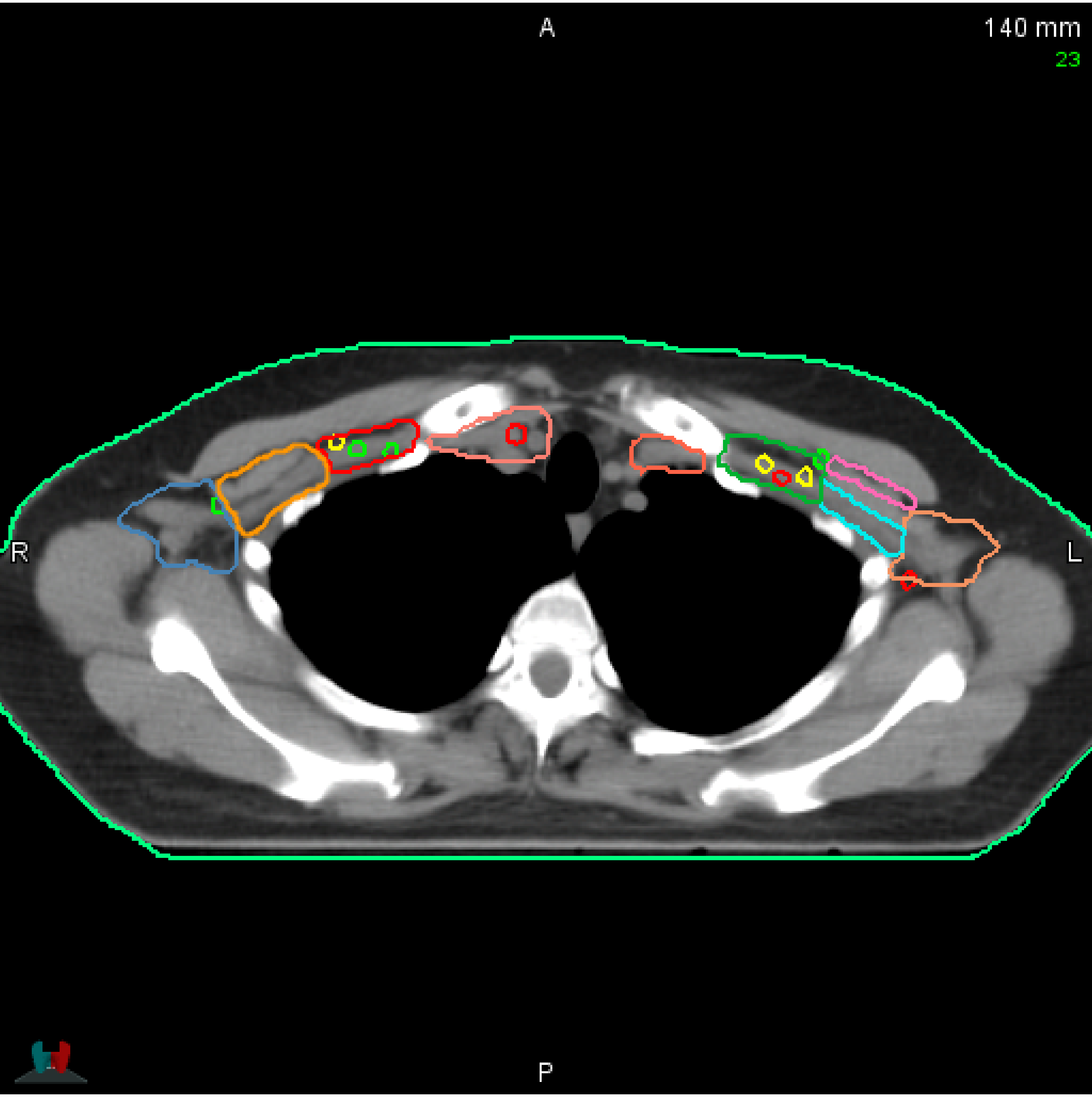
A
140 mm
23
R
L
P

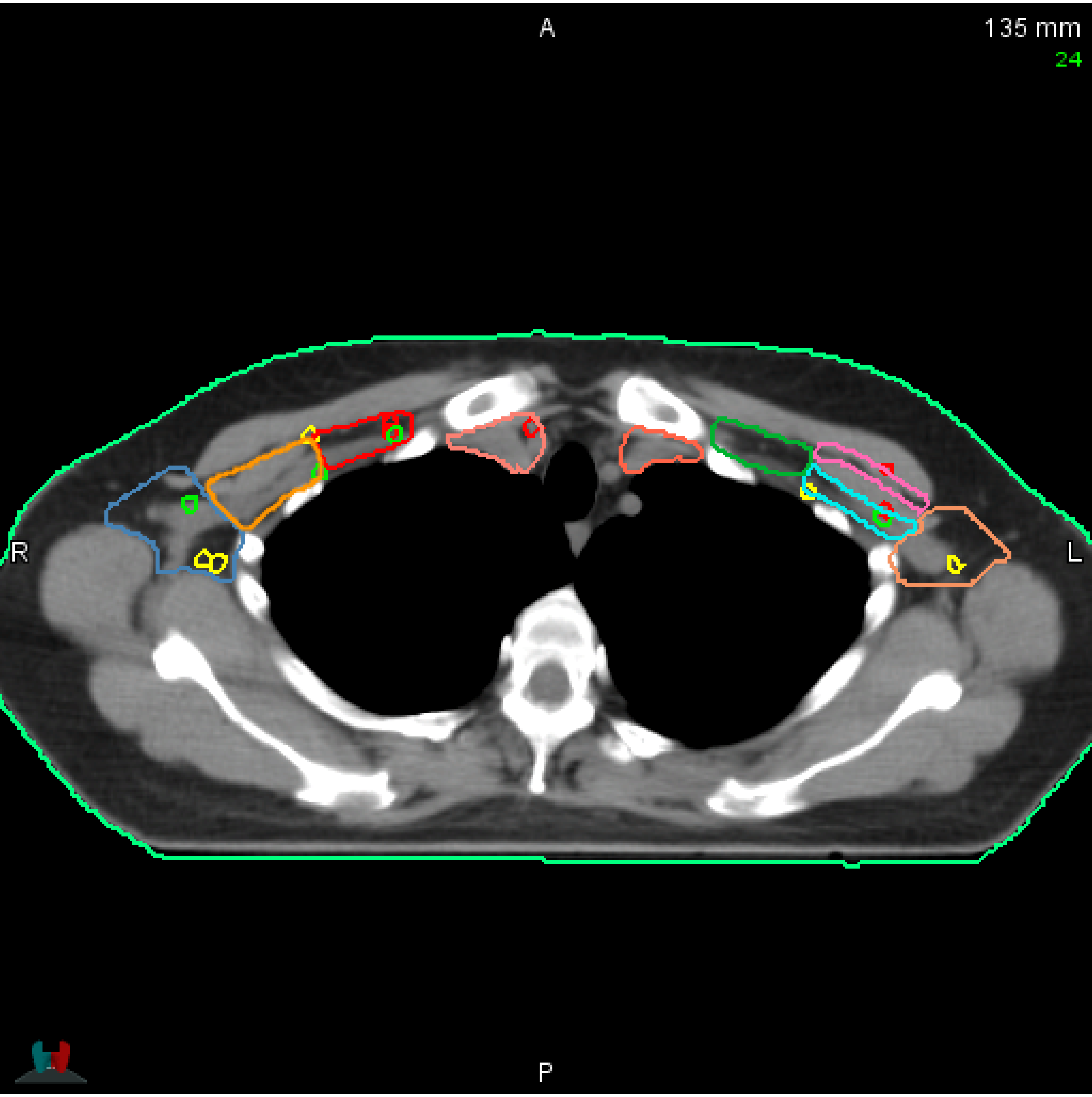

A
135 mm
24
R
L
P

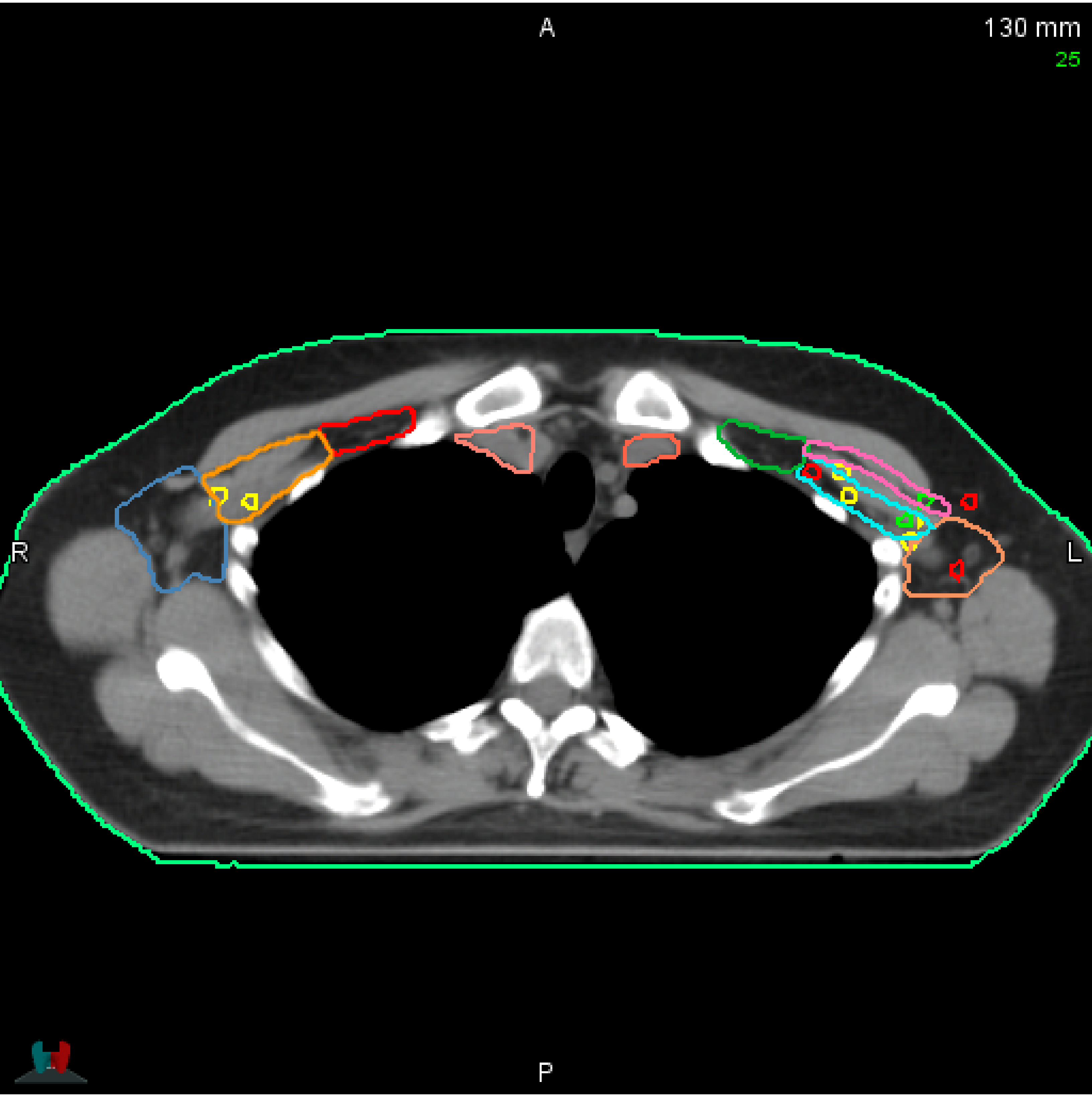
A
130 mm
25
R
L
P

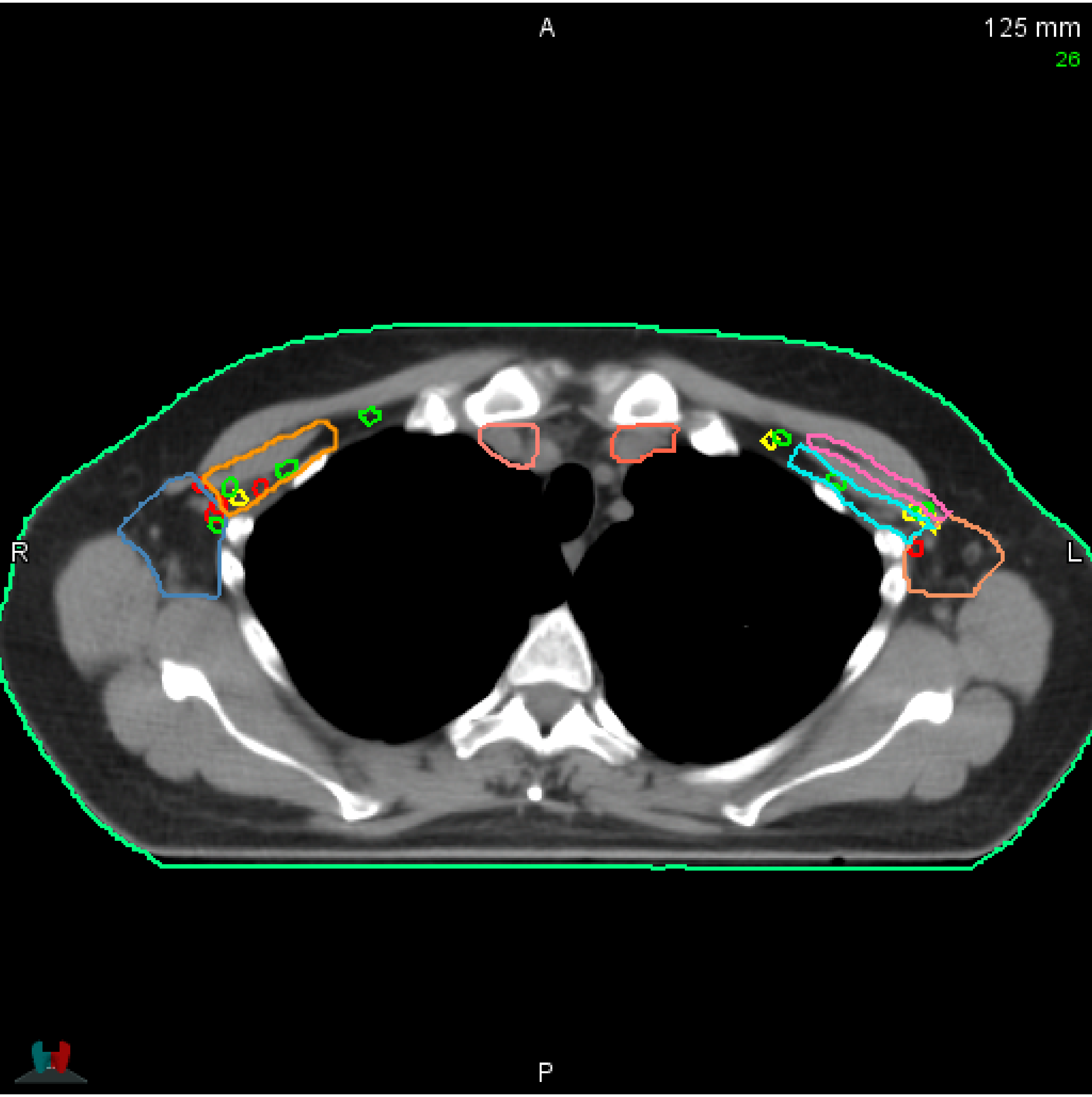
A
125 mm
26
R
L
P

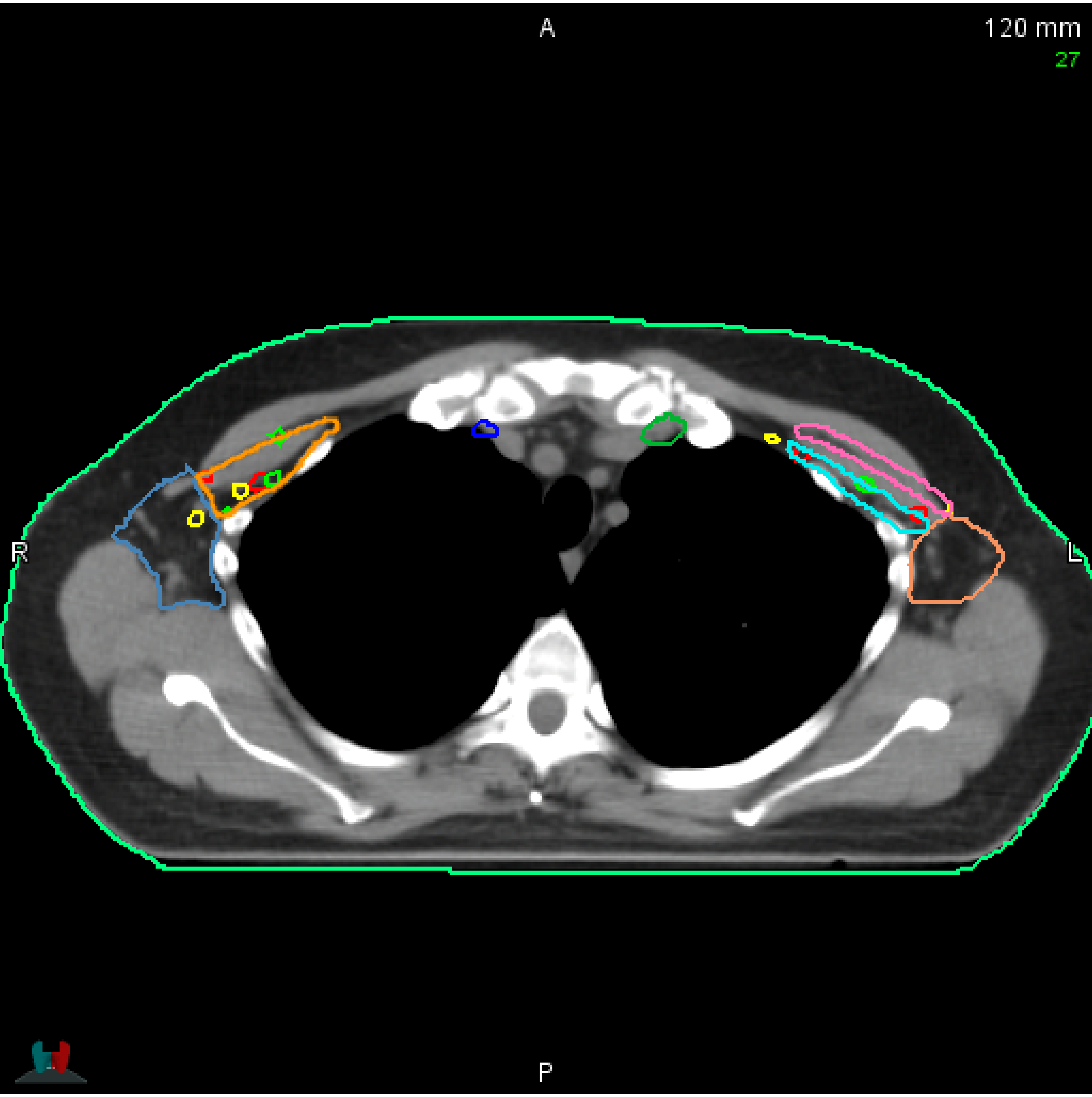
A
120 mm
27
R
L
P

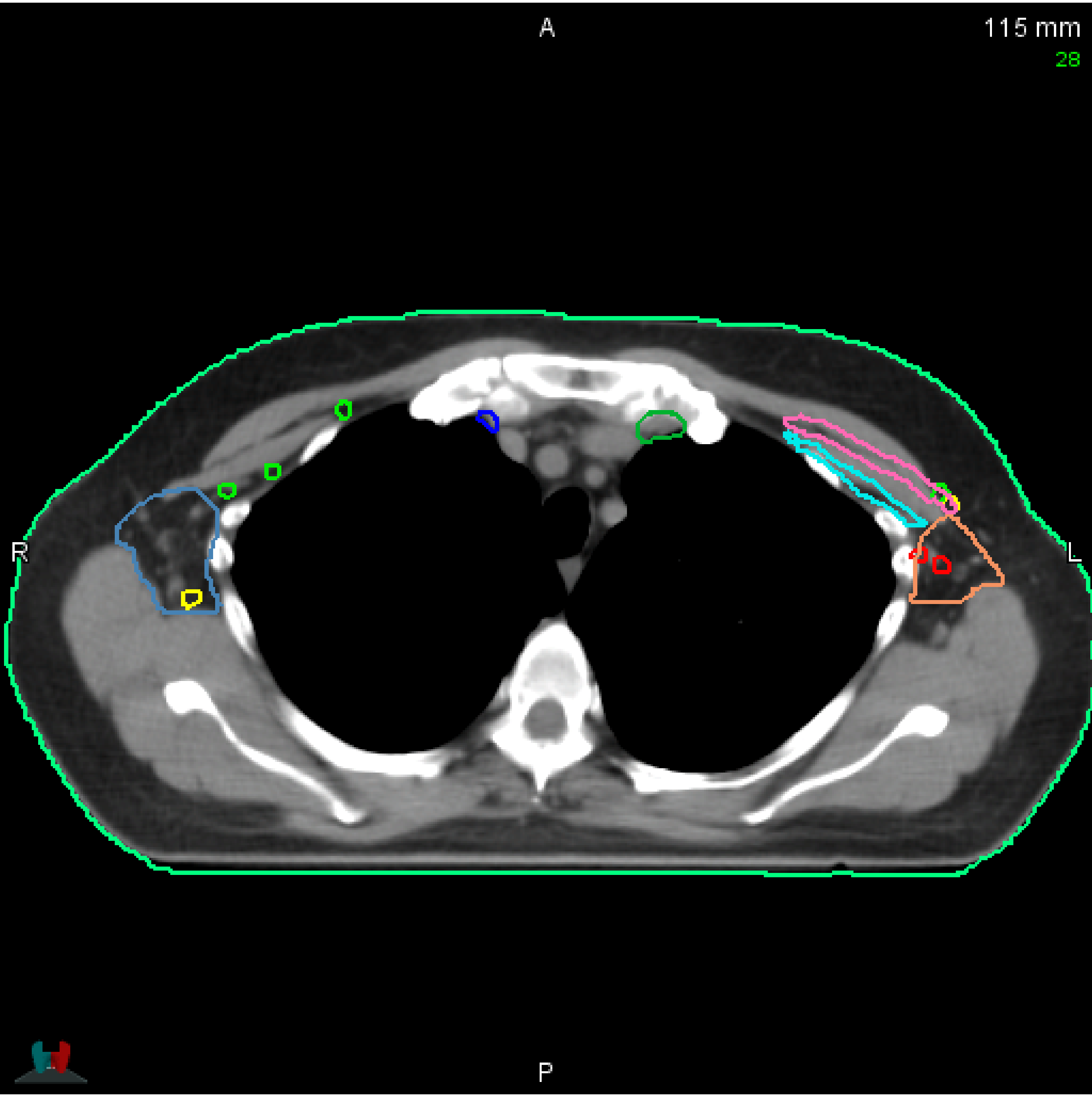
A
115 mm
28
R
L
P

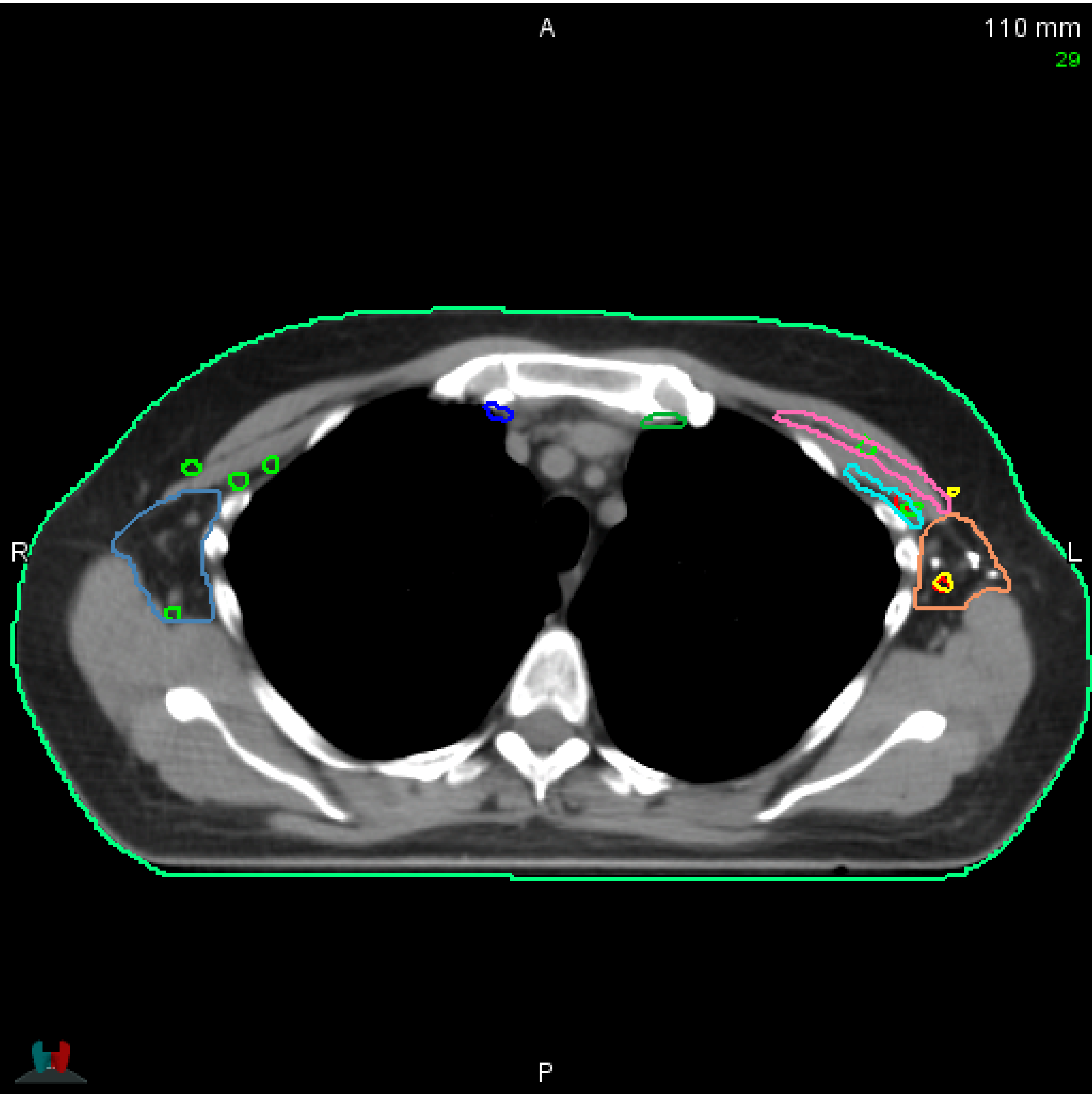
A
110 mm
29
R
L
P

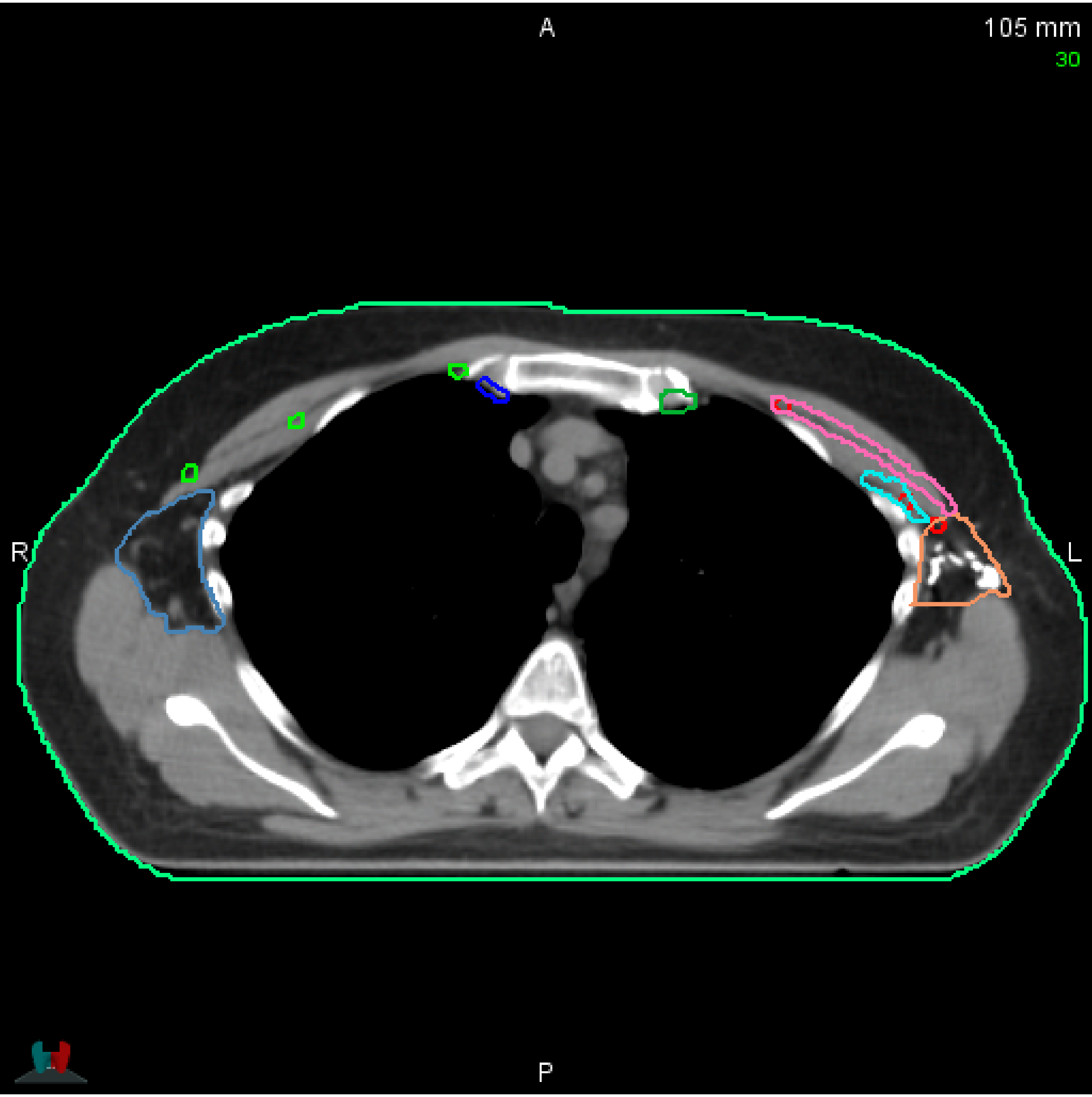
A
105 mm
30
R
L
P

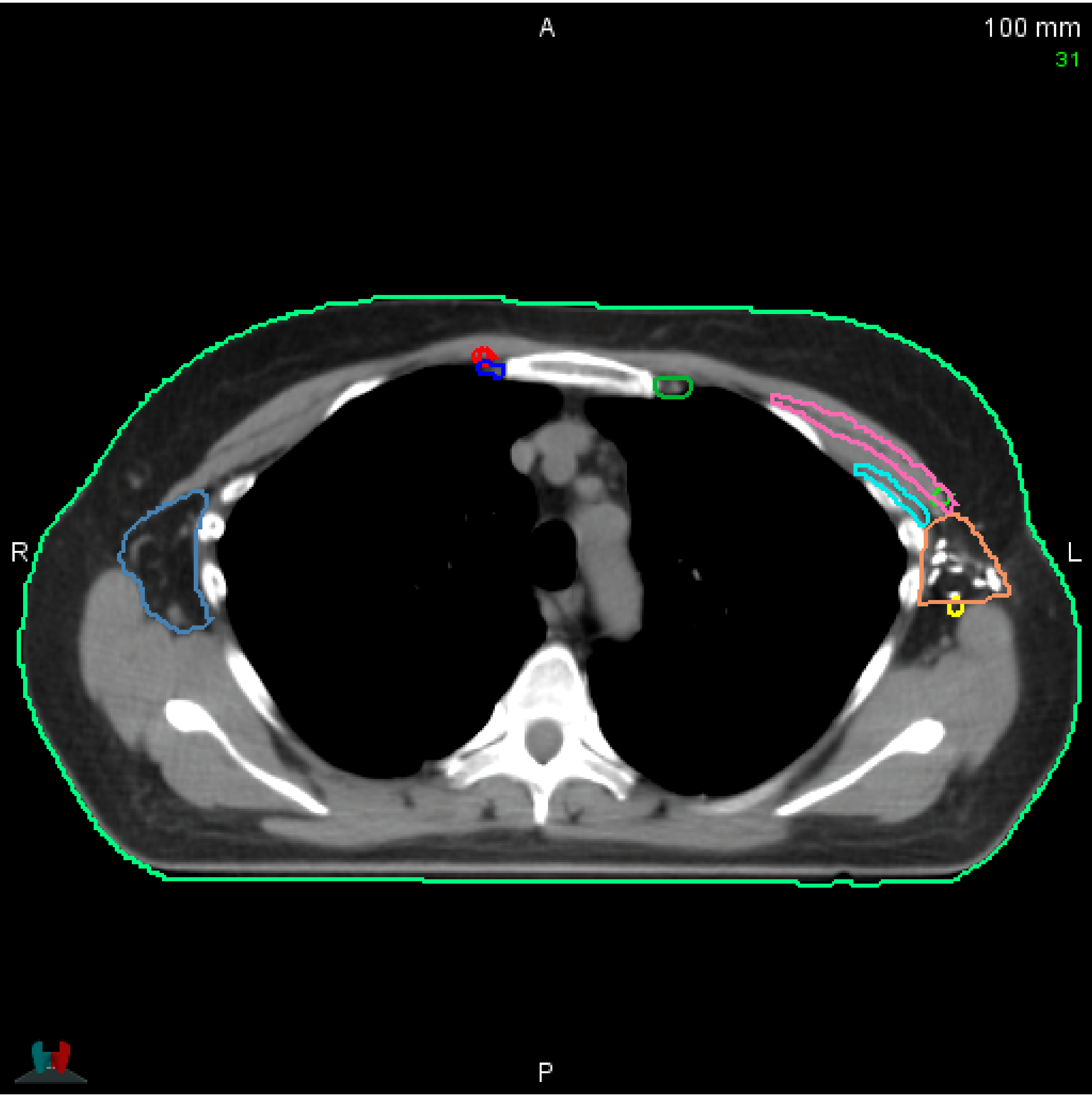
A
100 mm
31
R
L
P

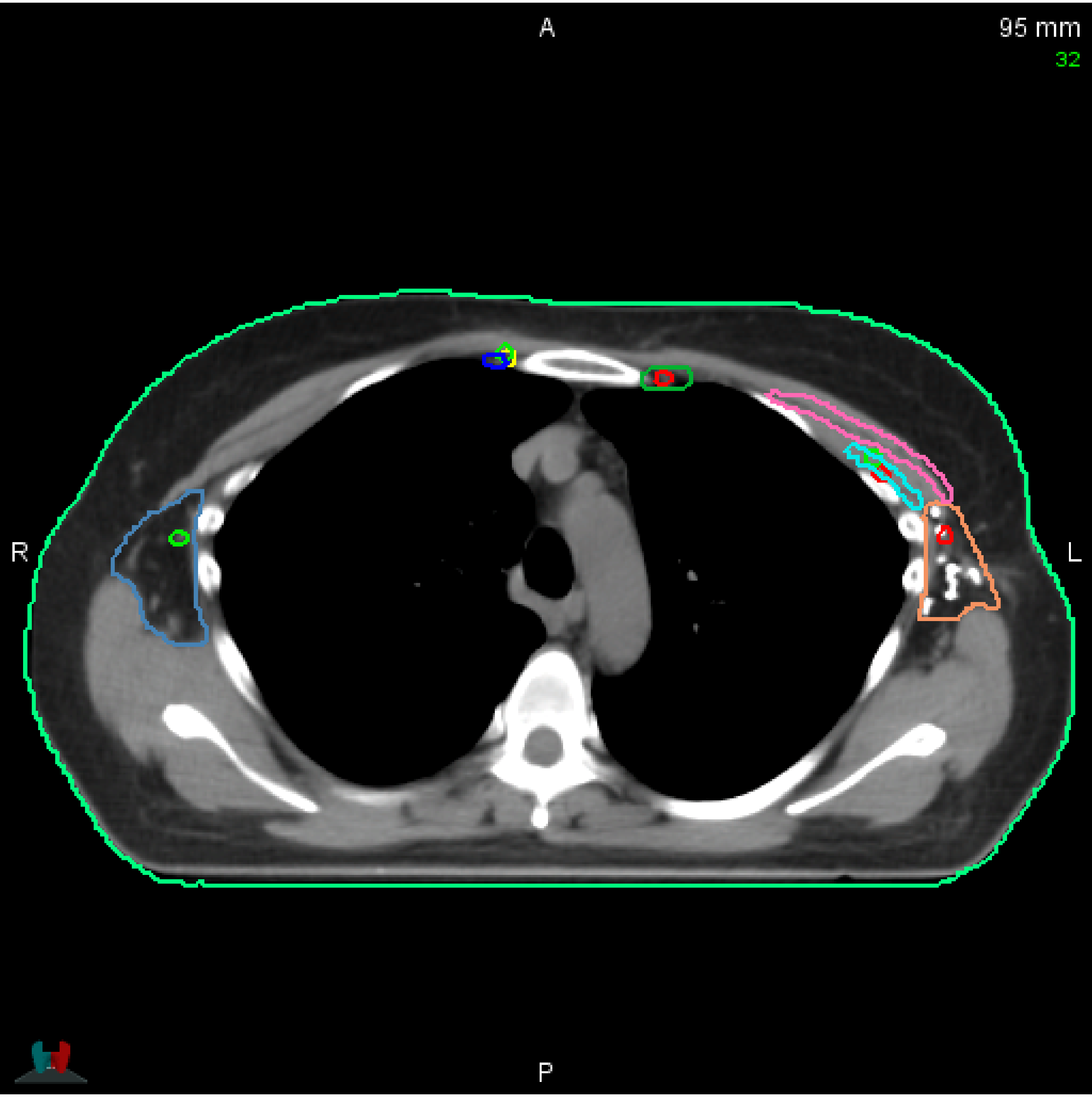
A
95 mm
32
R
L
P

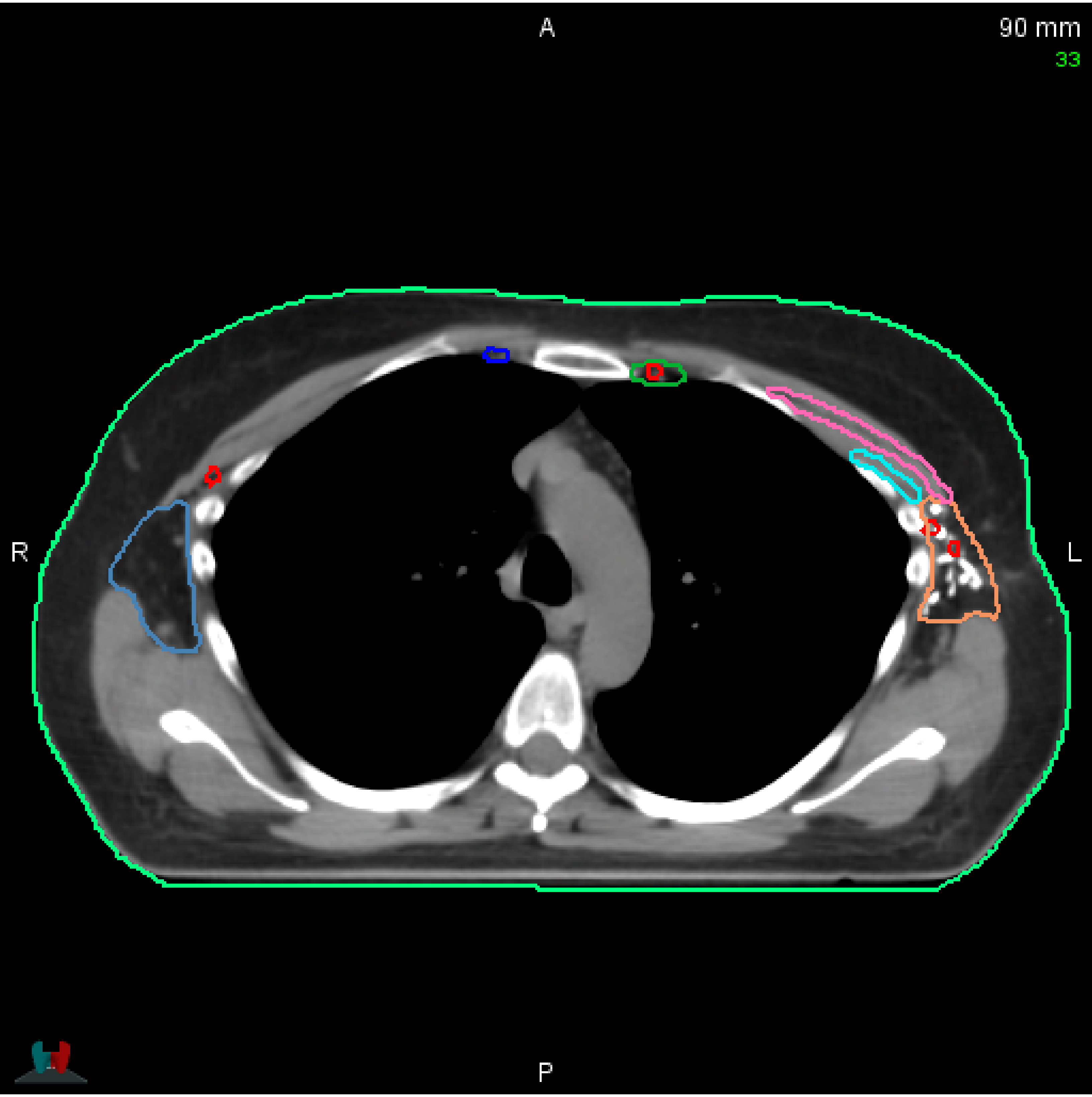

A
90 mm
33
R
L
P

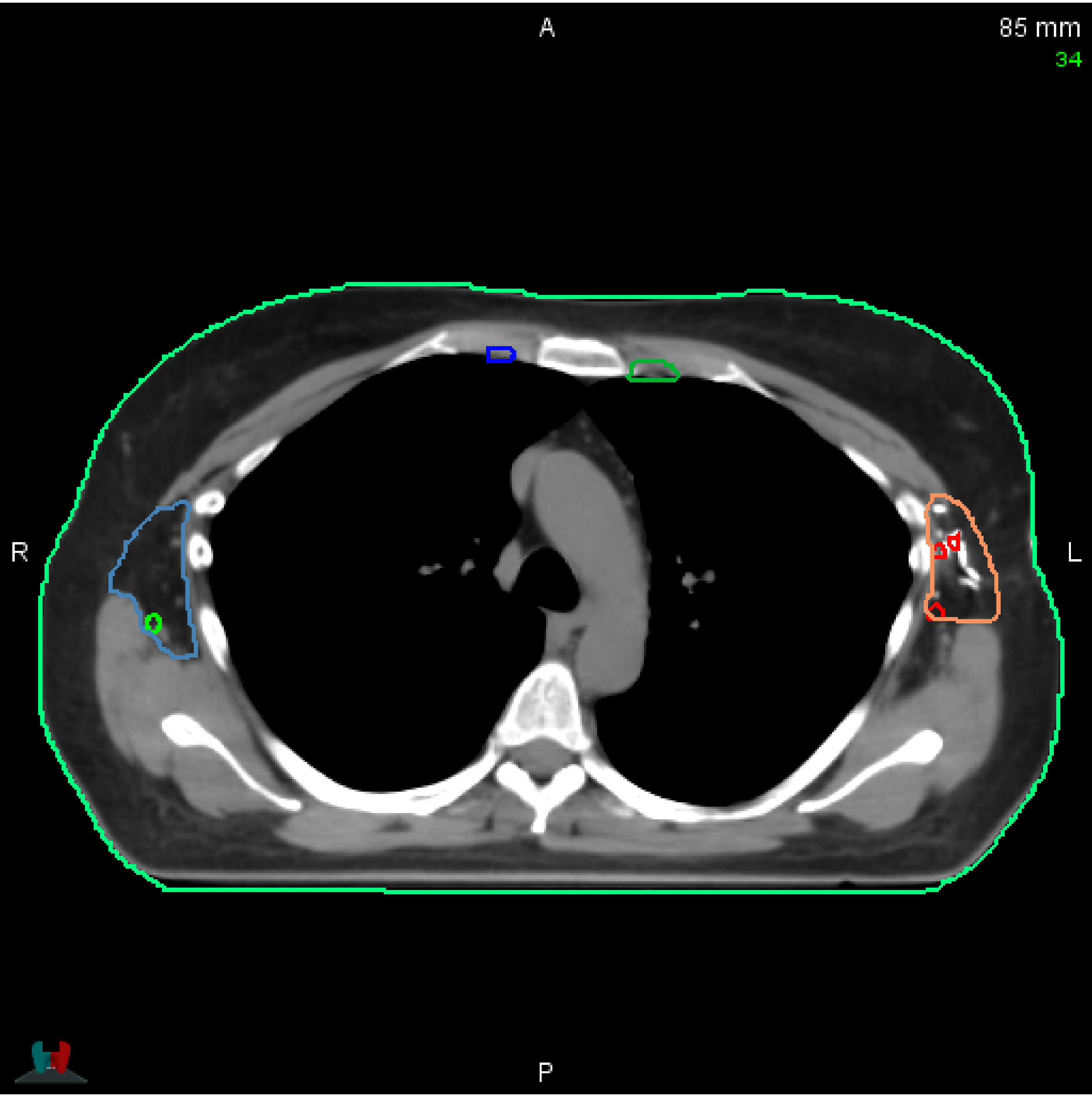
A
85 mm
34
R
L
P

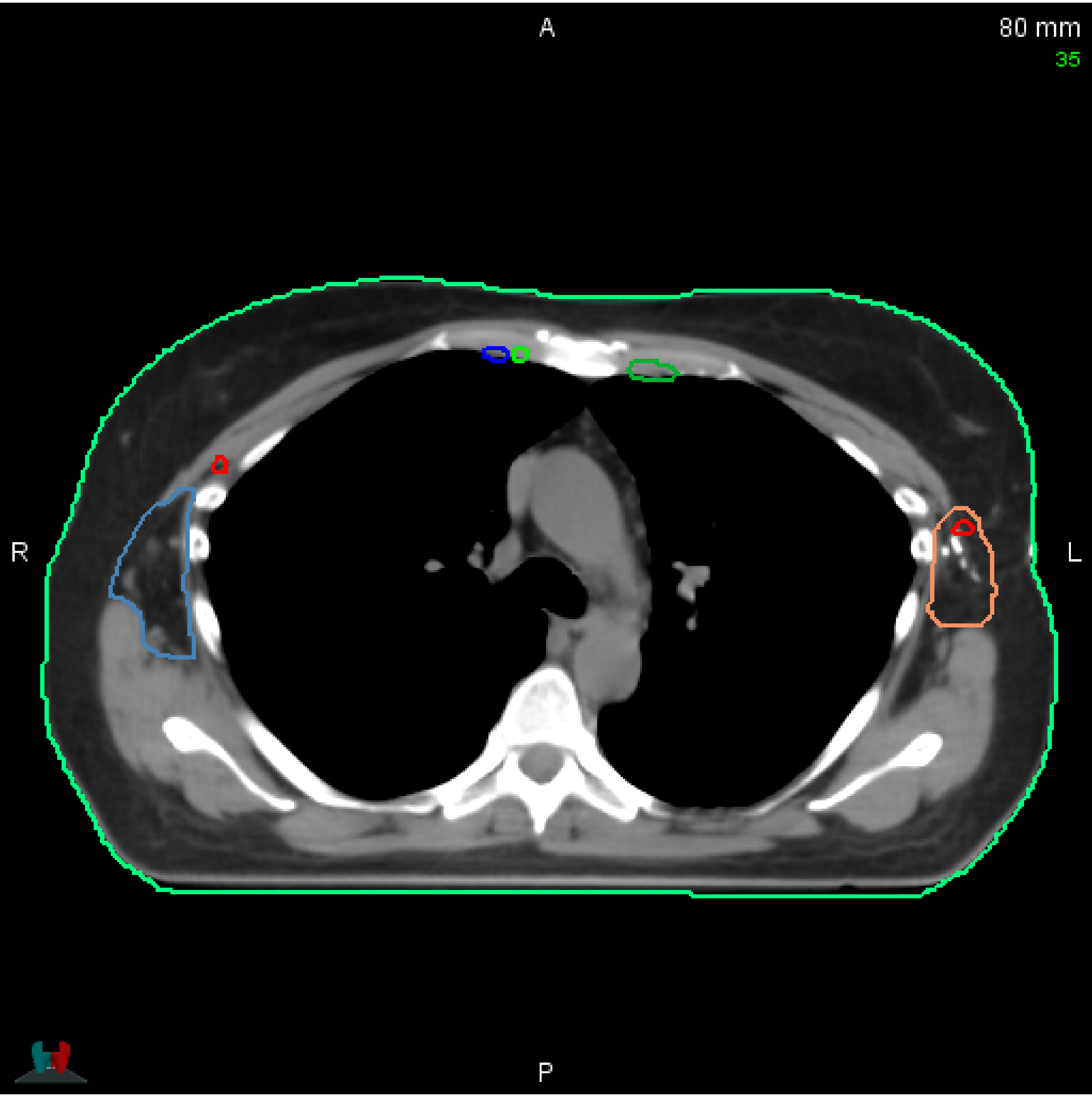
A
80 mm
35
R
L
P

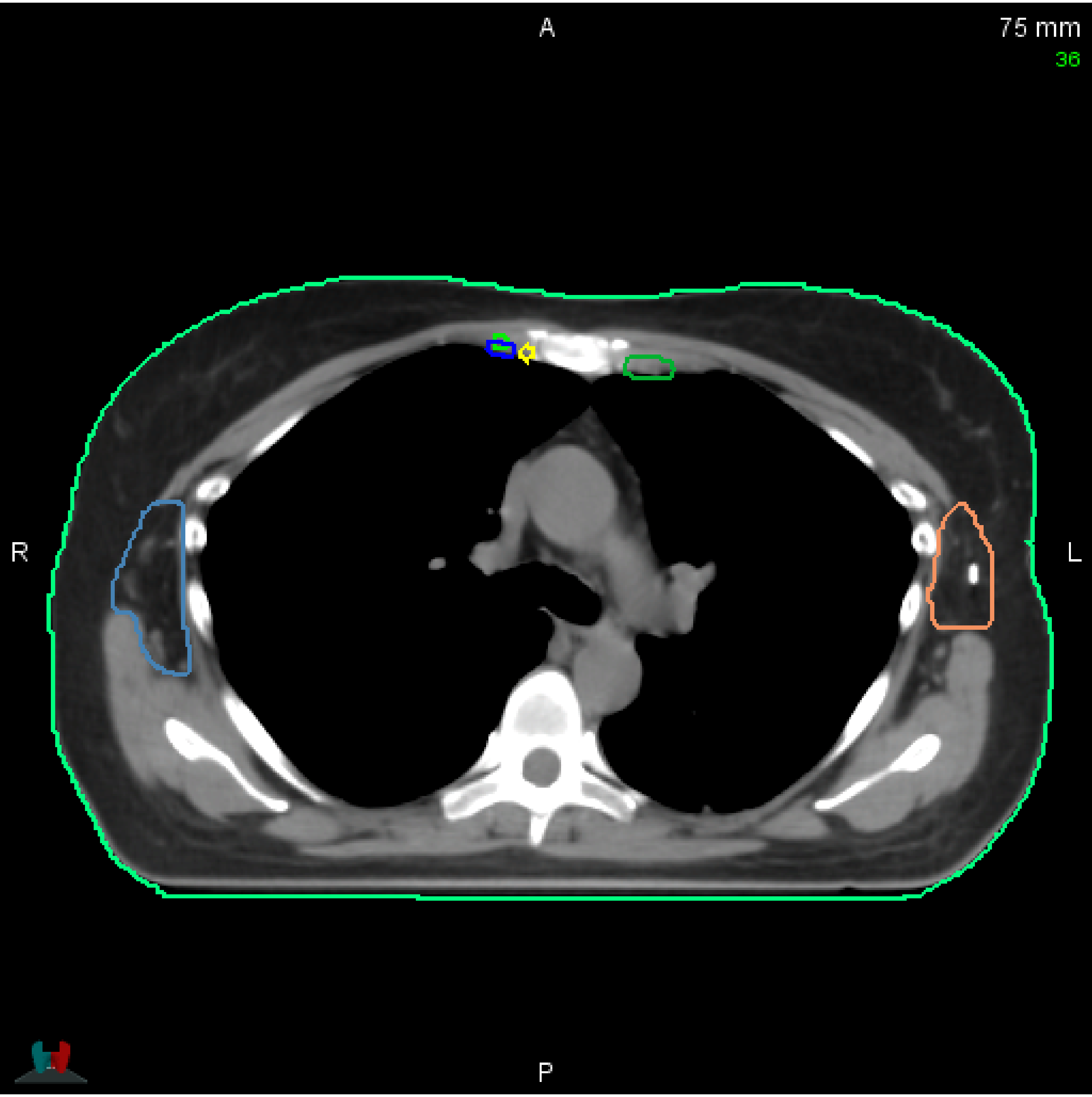
A
75 mm
36
R
L
P

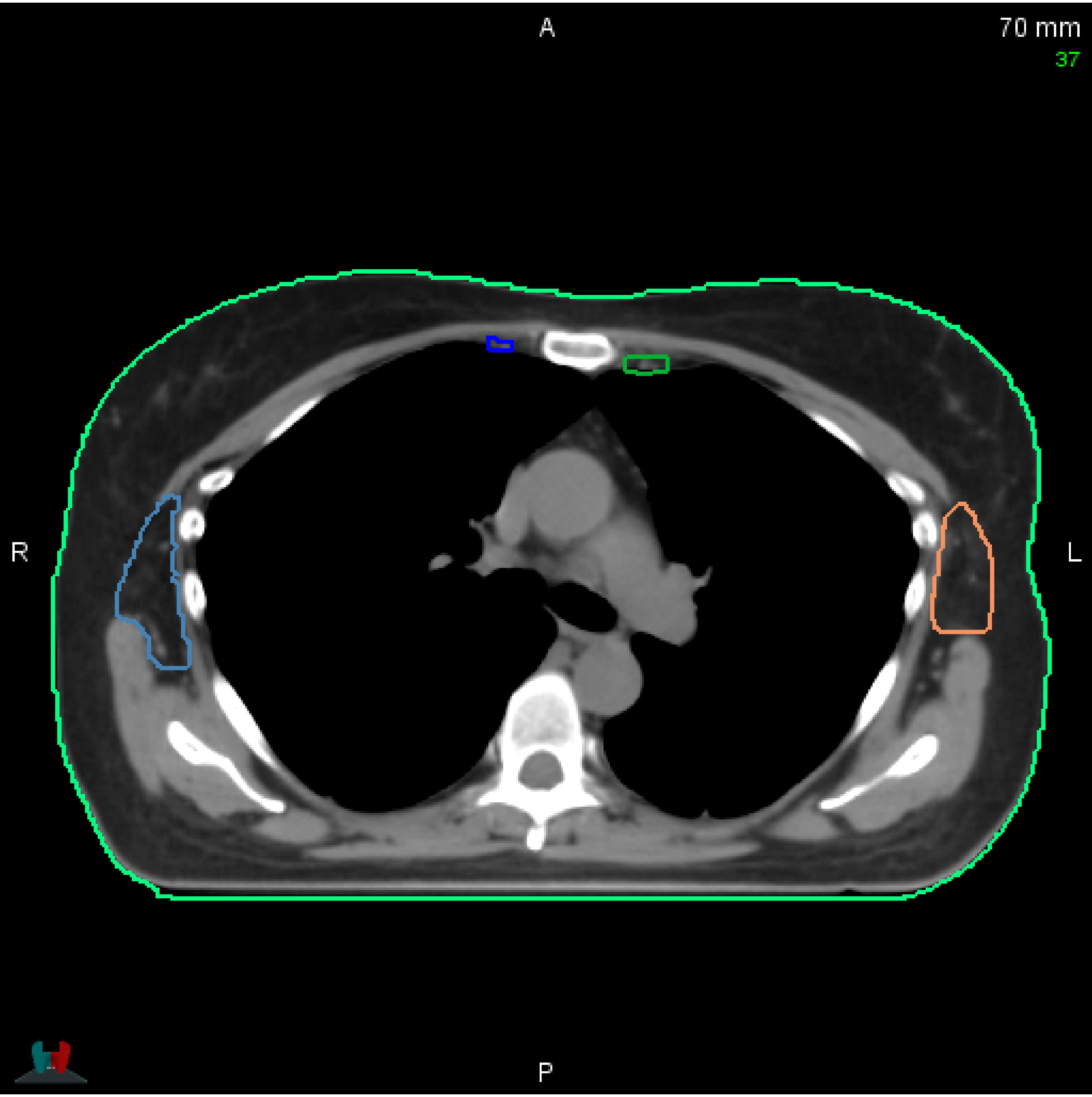
A
70 mm
37
R
L
P

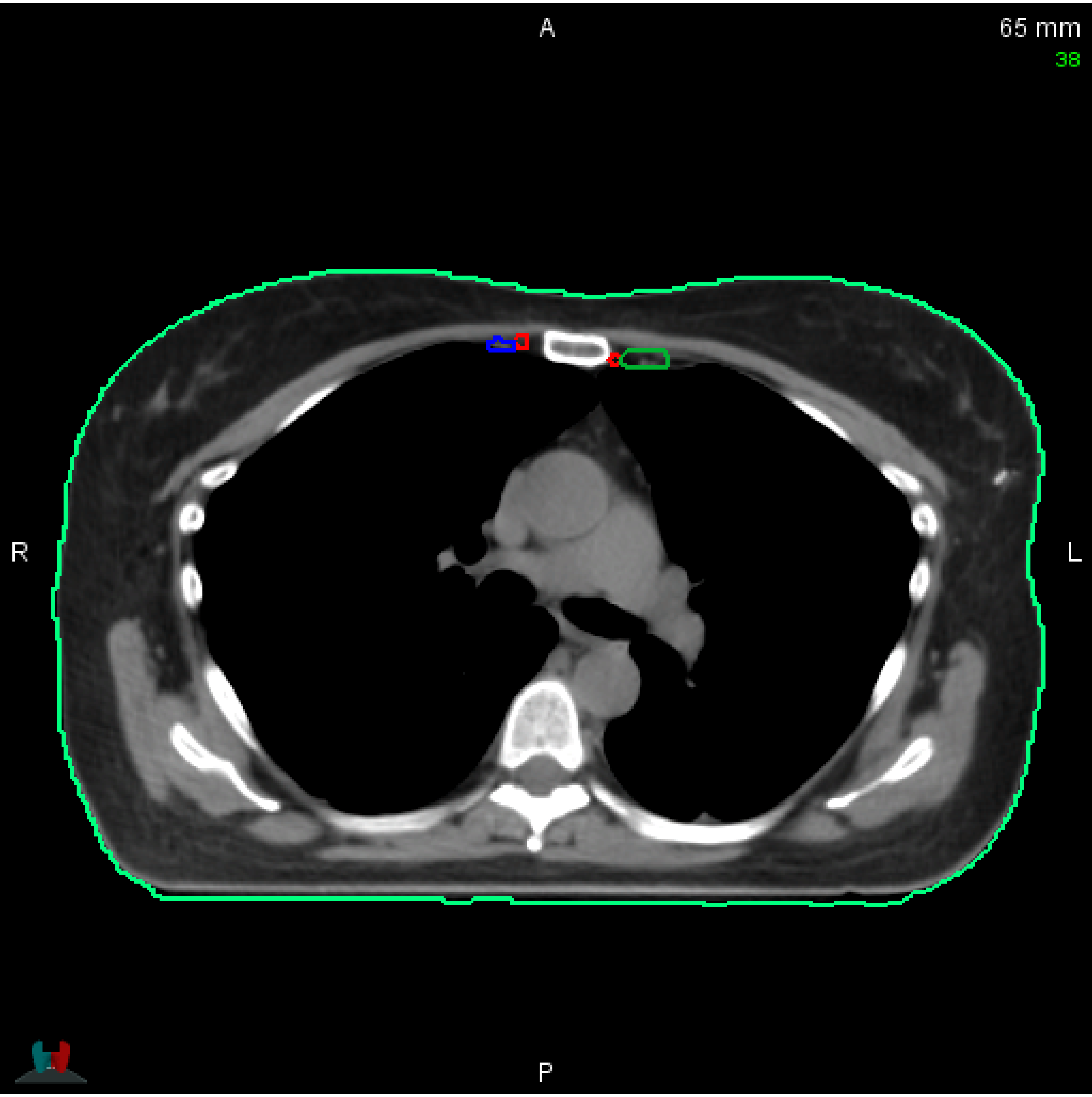
A
65 mm
38
R
L
P

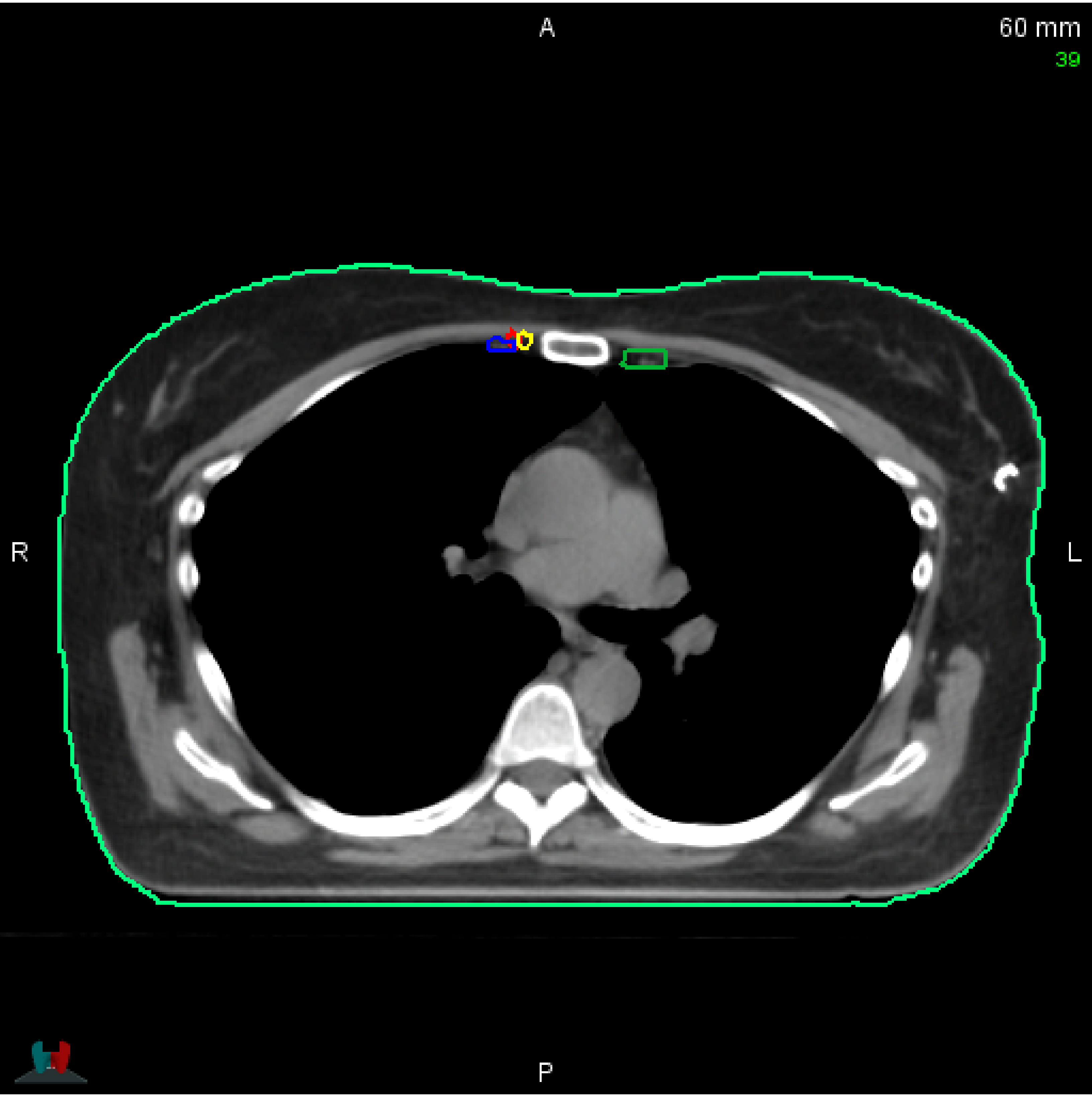
A
60 mm
39
R
L
P

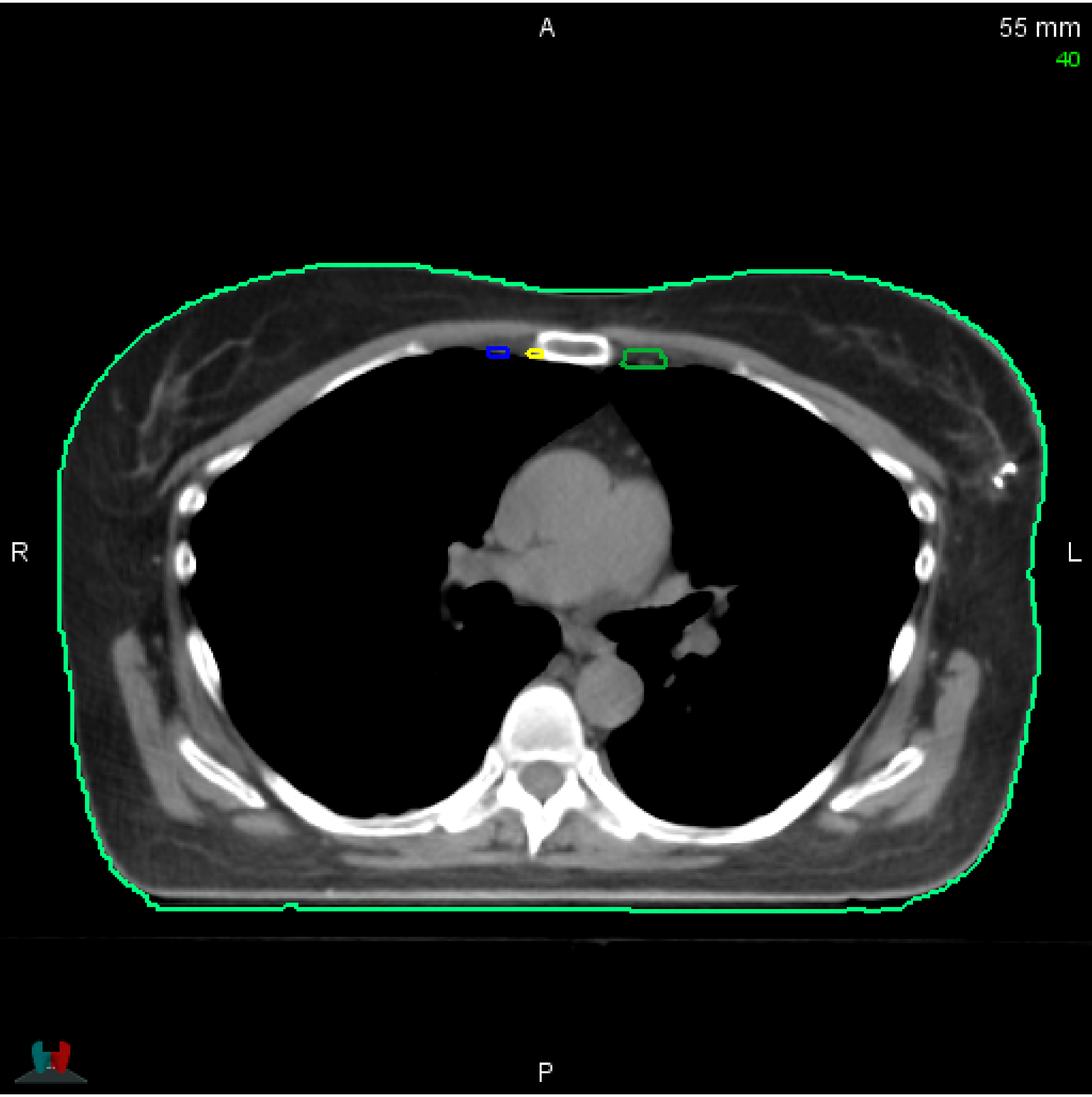
A
55 mm
40
R
L
P

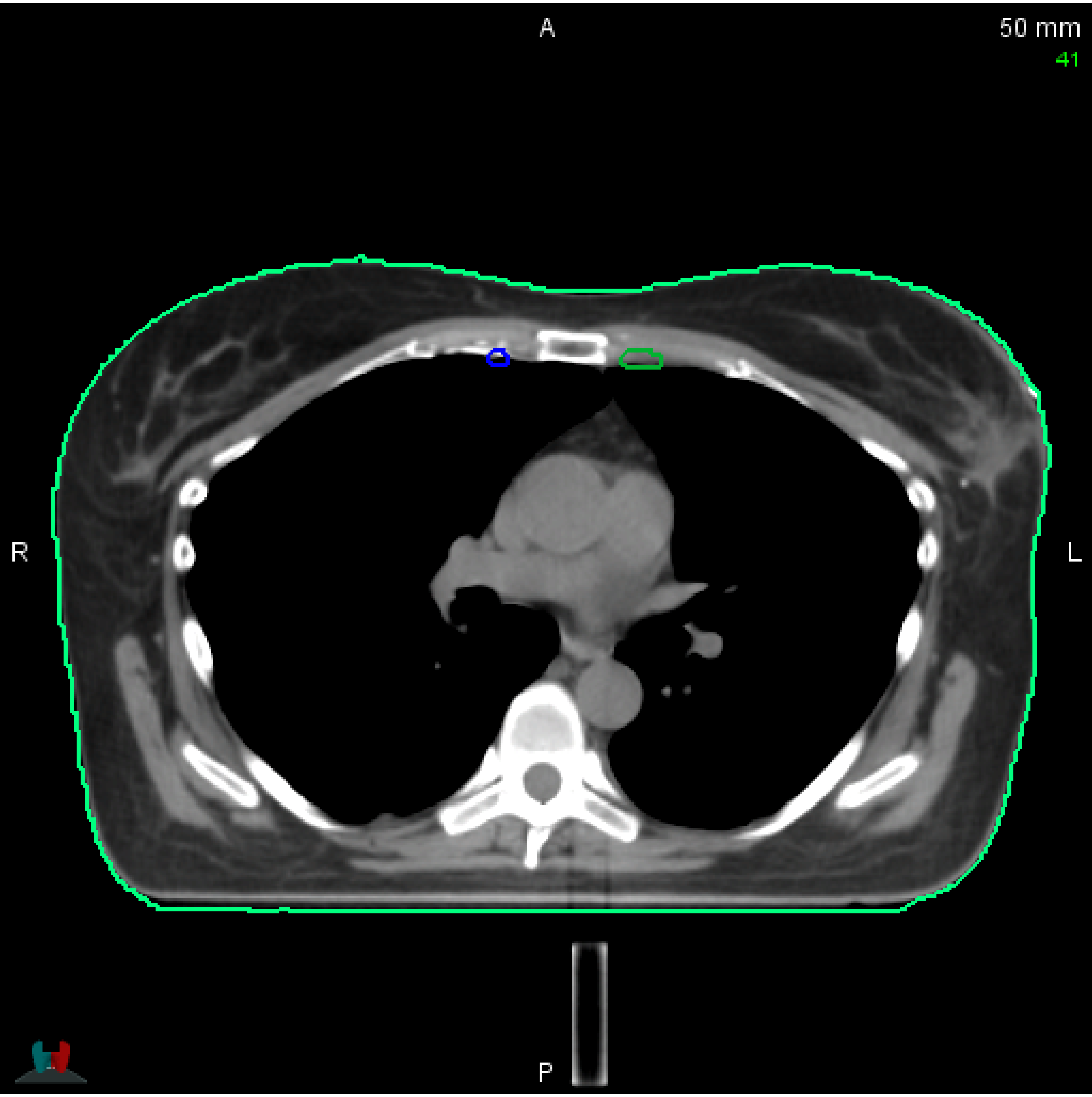
A
50 mm
41
R
L
P

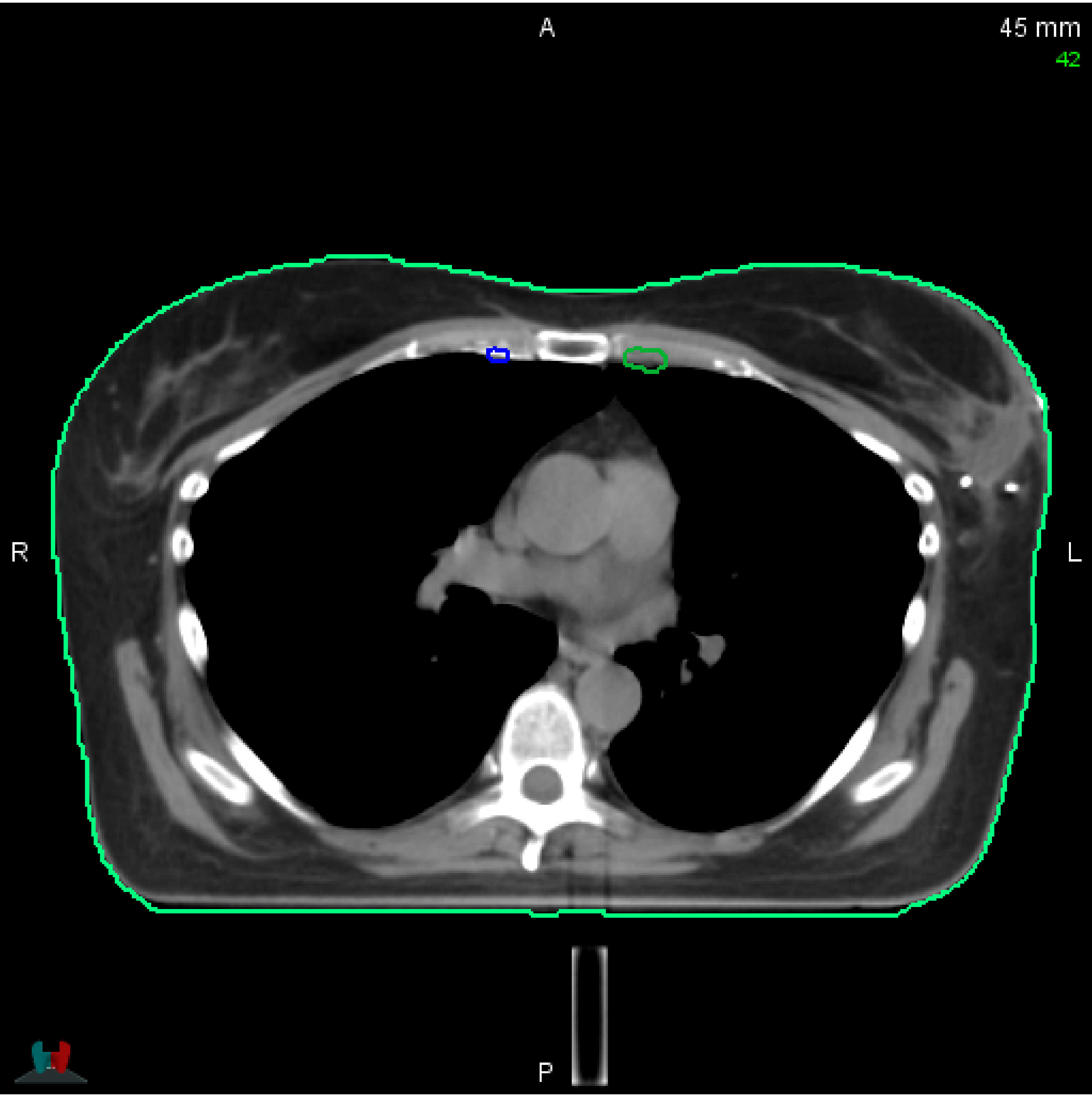
A
45 mm
42
R
L
P

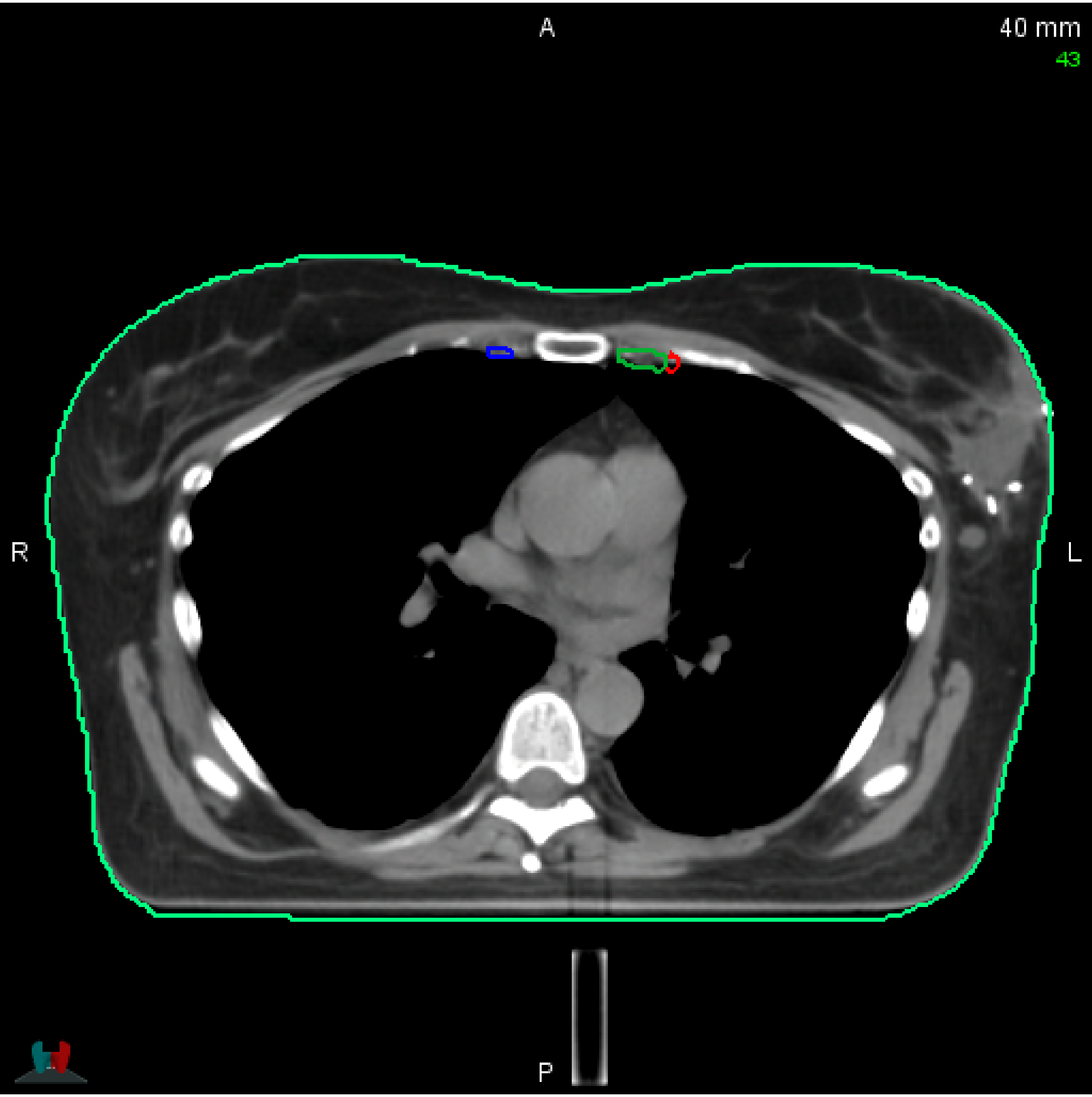

A
40 mm
43
R
L
P

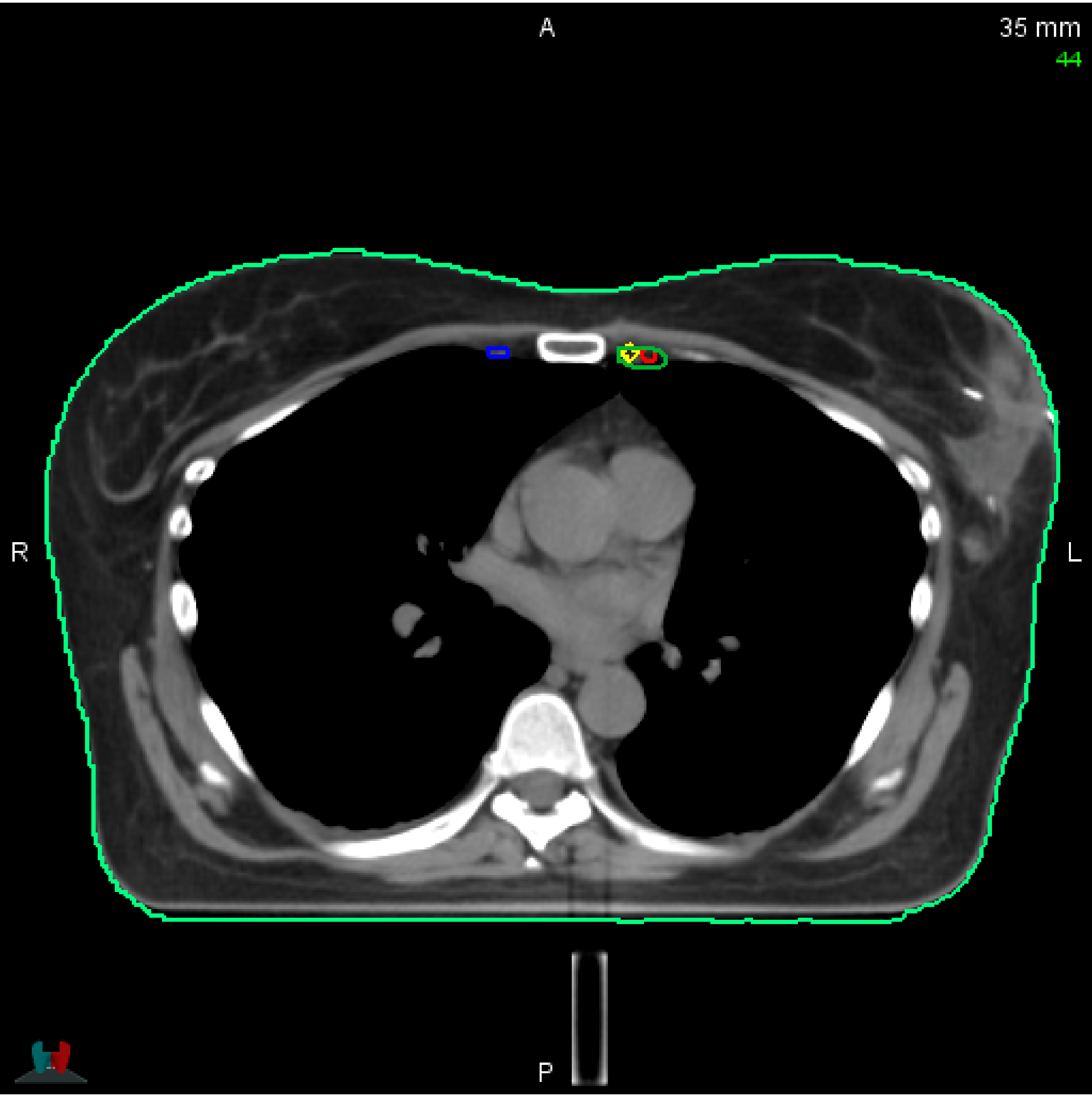

A
35 mm
44
R
L
P

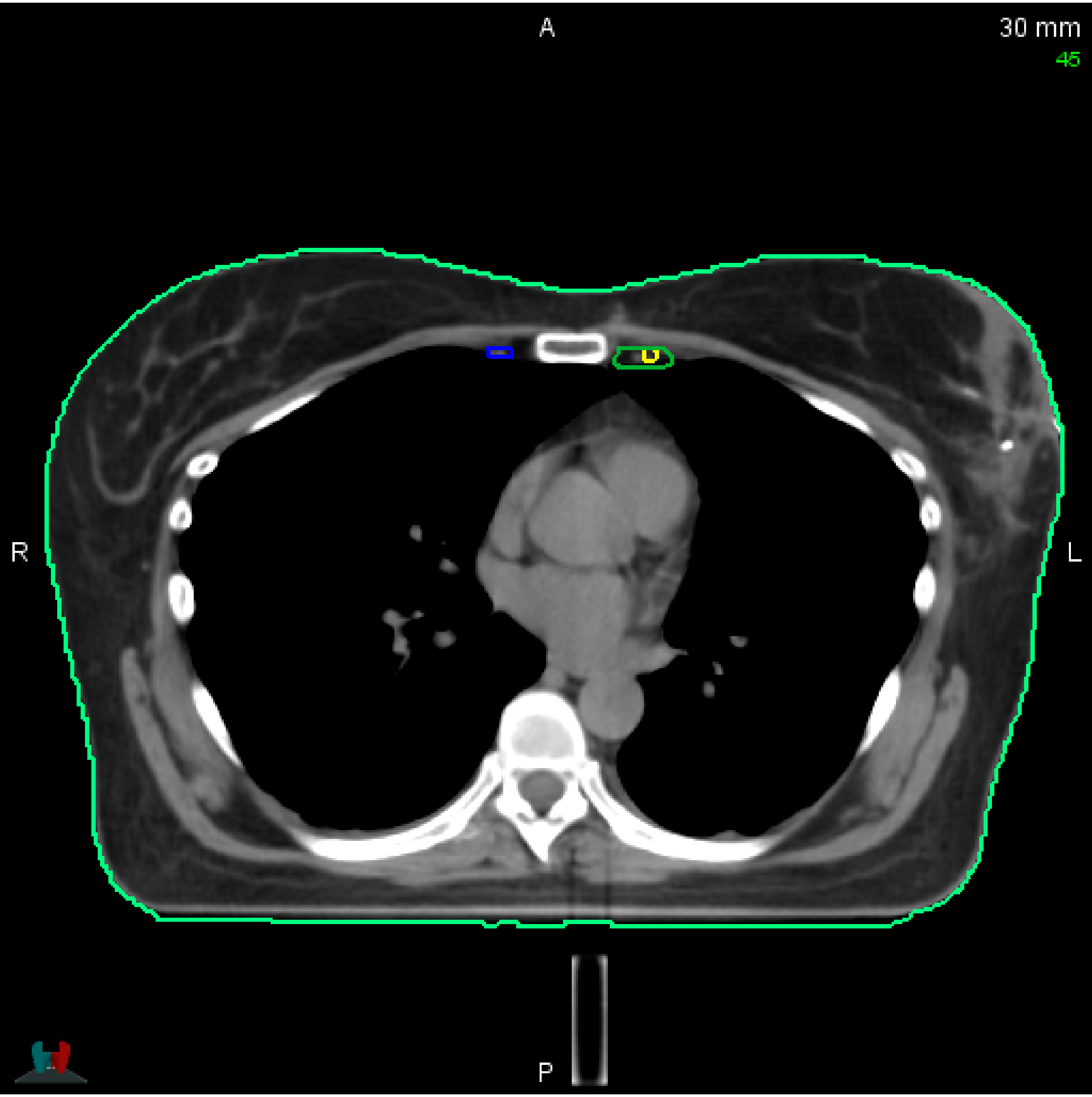
A
30 mm
45
R
L
P

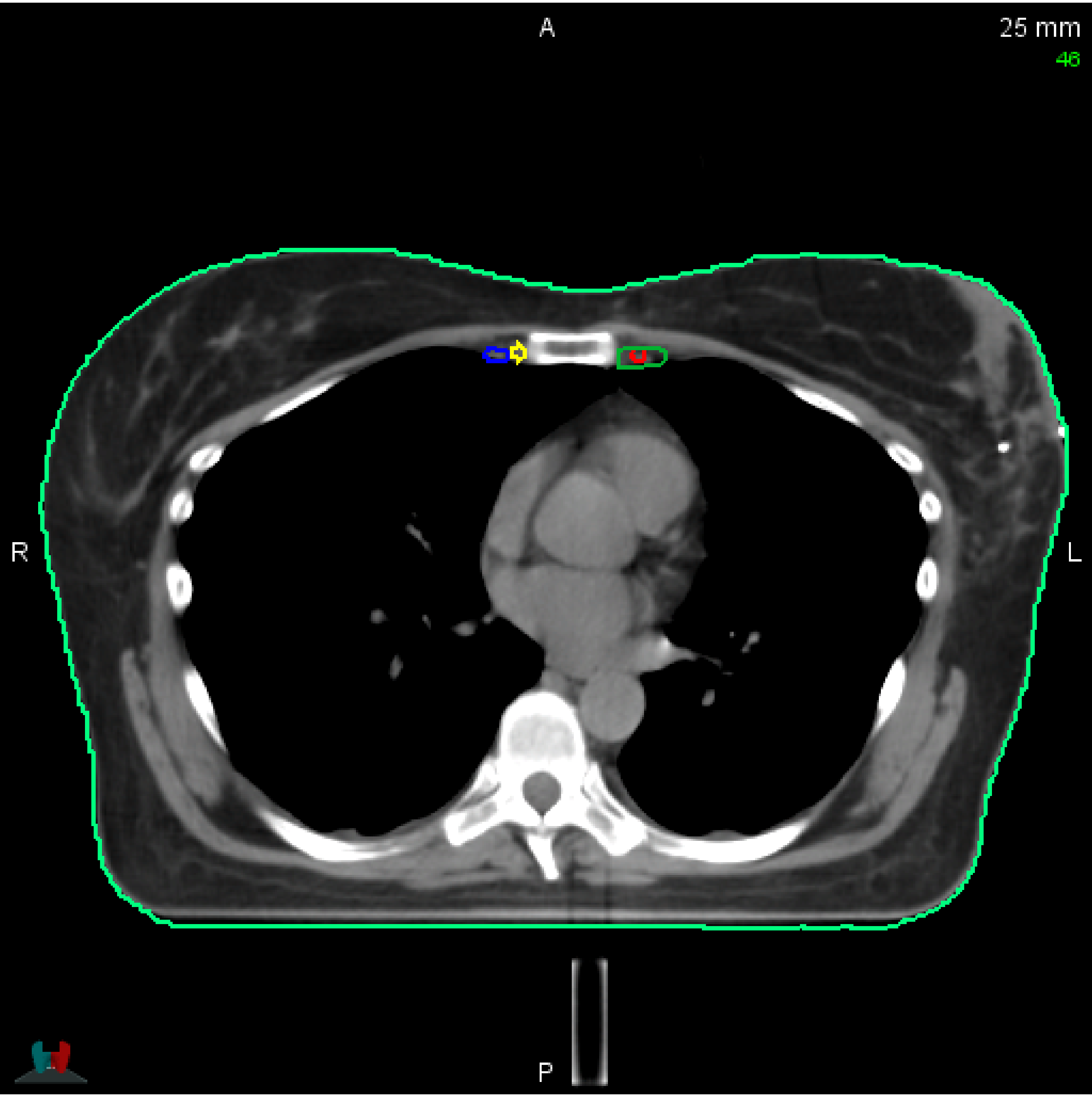
A
25 mm
46
R
L
P

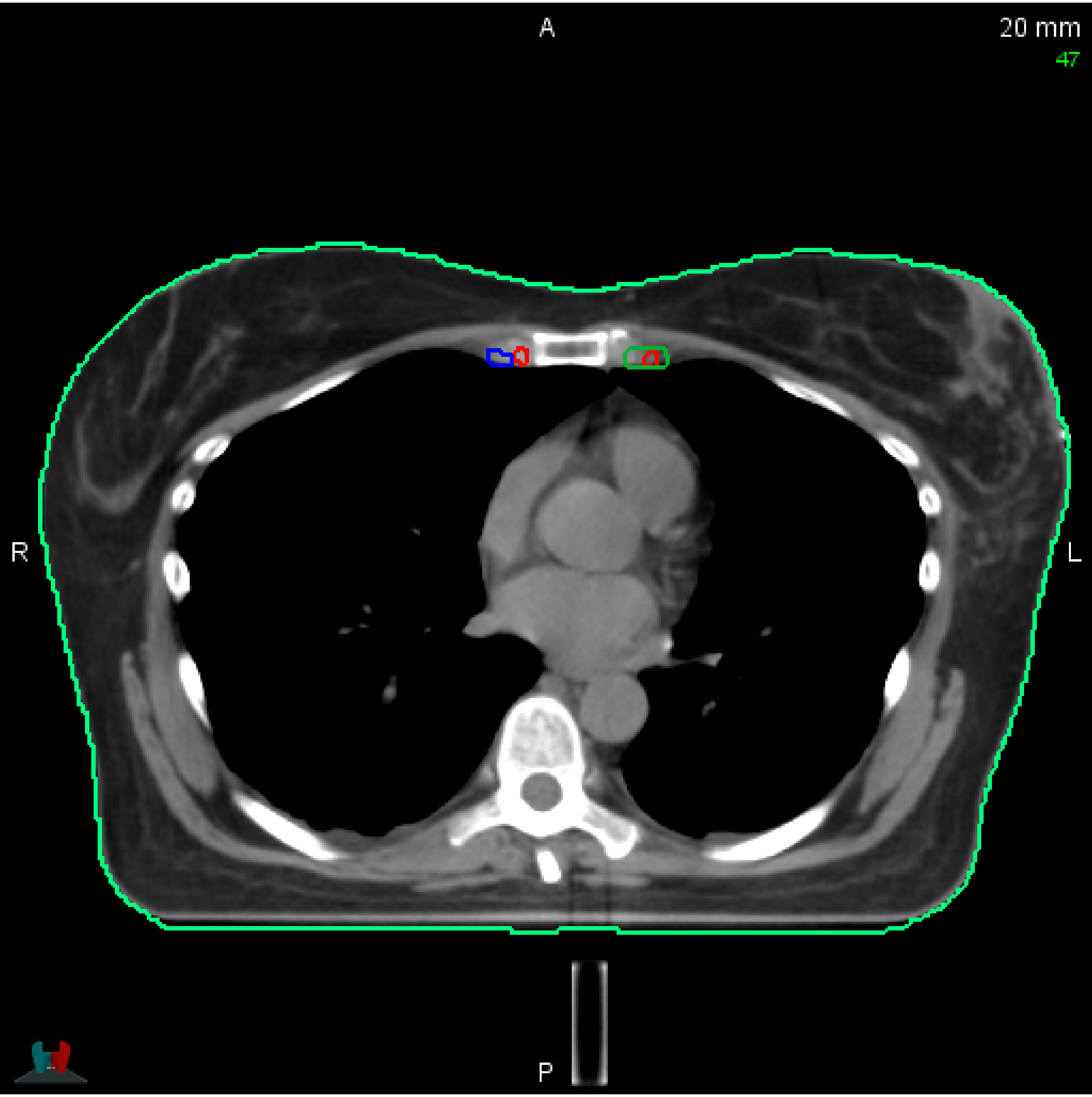
A
20 mm
47
R
L
P

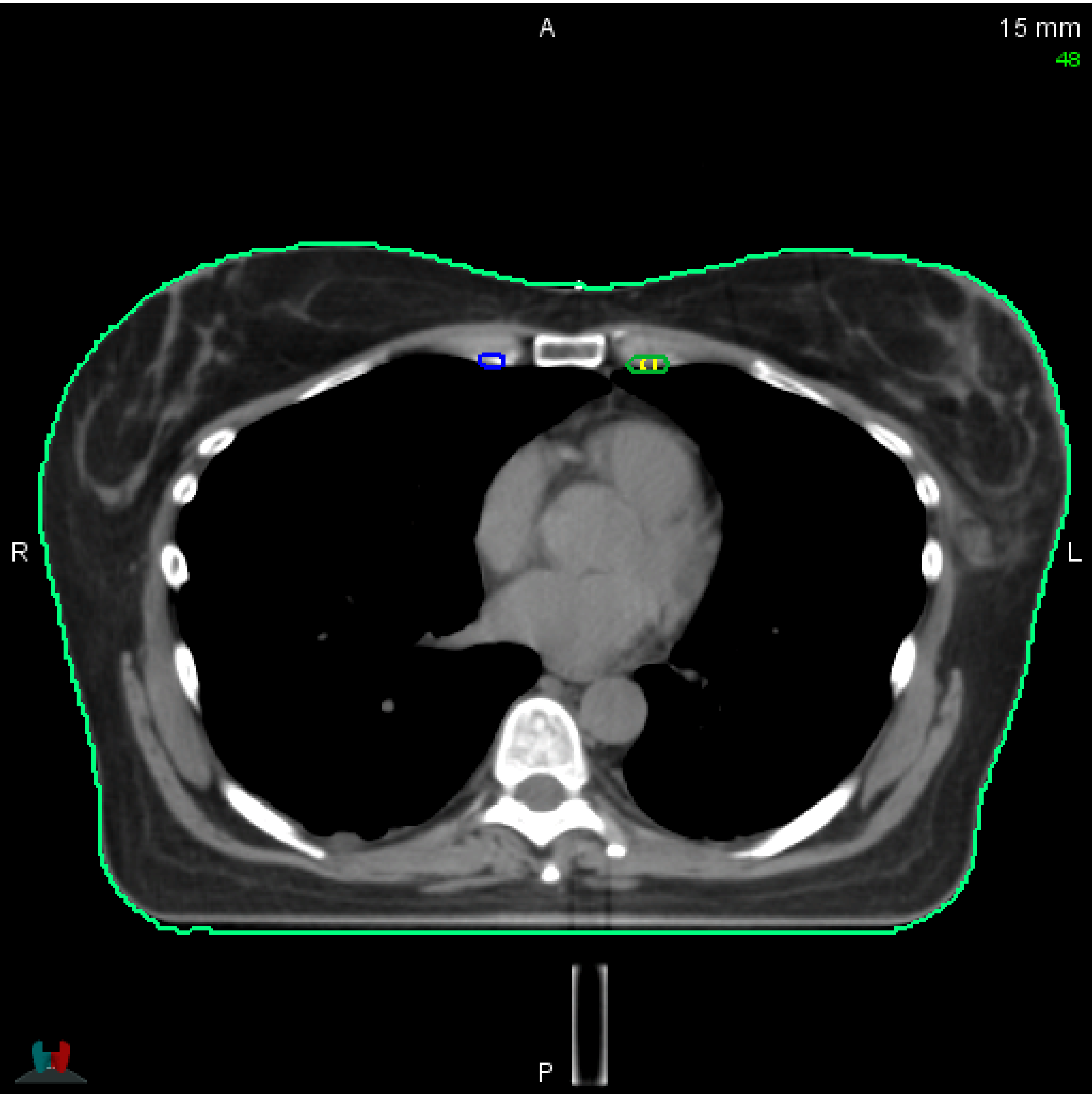
A
15 mm
48
R
L
P

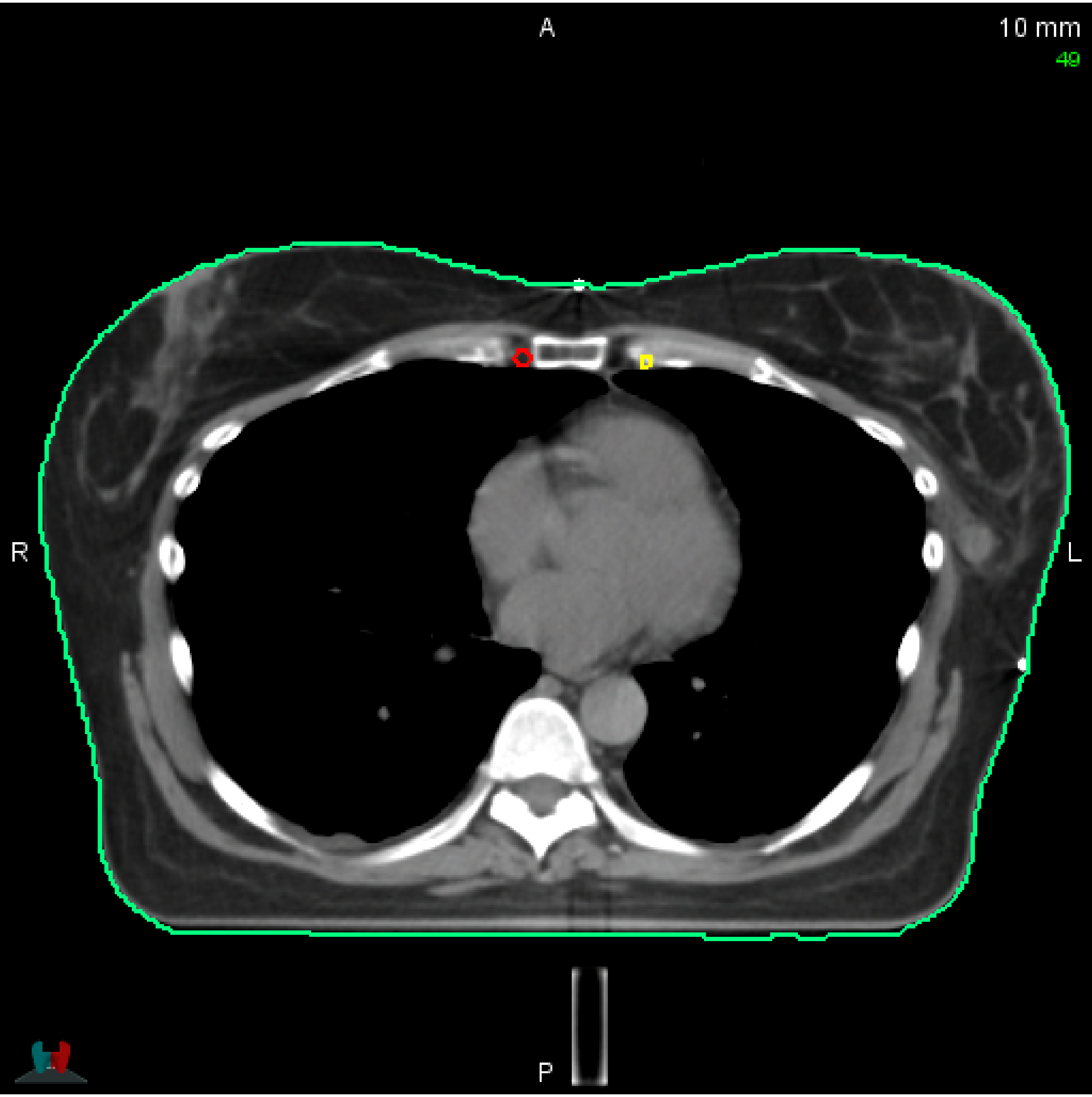

A
10 mm
49
R
L
P
